# Soil algae in arable land: changes in the genotypic community composition across time points and farming systems—a pilot study

**DOI:** 10.3389/fmicb.2026.1813833

**Published:** 2026-04-16

**Authors:** Stefan Barthel, Miriam Athmann, Tatyana Darienko, Nataliya Rybalka, Birgit Olberg, Maike Lorenz, Thomas Friedl

**Affiliations:** 1 Department Experimental Phycology and Culture Collection of Algae (EPSAG), Albrecht-von-Haller-Institute for Plant Sciences, Georg August University, Göttingen, Germany; 2 Section of Organic Farming and Cropping Systems, University of Kassel, Witzenhausen, Germany

**Keywords:** arable land, Chlorophyta, Cyanobacteria, farming system, genotypic community composition, microbial diversity, soil algae, Stramenopiles

## Abstract

**Introduction:**

Soil algae, including Cyanobacteria, in the topsoils of arable land are essential for many valuable effects on soil texture and fertility. However, the constant changes and disturbances caused by agricultural practices create a challenging environment. We tested for responses in soil algal diversity across principal time points during the growth of *Triticum aestivum*, a typical winter cereal under Central European agricultural practices, and examined the effects of contrasting farming systems.

**Methods:**

We employed a metabarcoding analysis based on amplicons from multiple markers to assess changes in the genotypic community composition.

**Results and discussion:**

Relationships among the detected key groups of soil algae, i.e., Chlorophyta (Chlorophyceae, Trebouxiophyceae), Stramenopiles (Xanthophyceae, Diatomeae), and Cyanobacteria, varied significantly across the three principal time points of the wheat’s growth season. The eukaryotic algae (118 genera) alone accounted for approximately one-third in March to approximately one-half in July of all microeukaryote ASVs in the surface soils. The 23S UPA marker, used to amplify eukaryotic algae and Cyanobacteria in a single step, and the 18S marker differed in their estimates of ASV numbers for Chlorophyta and Xanthophyceae. However, in combination with markers specifically targeting the green algal ITS2 and the *rbc*L genes of Stramenopiles and Chlorophyta, consistently the same distinct distribution patterns of the soil algal genotypes were found. Stramenopile genotypes were preferentially found in March and November at cold and high bare-soil availability, whereas Chlorophyta genotypes were most abundant in July at higher temperatures and minimal bare-soil availability. No Klebsormidiophyceae were found in March. The conventional management system favored algal biomass and ASV richness of Xanthophyceae, Diatomeae, and the green algal classes Ulvophyceae and Klebsormidiophyceae compared with organic farming. It negatively influenced the diversity of Cyanobacteria. Within conventional farming, *Zea mays*, as another crop, significantly increased the ASV richness of Chlorophyceae and Ulvophyceae, whereas that of Cyanobacteria decreased. A large fraction of genotypes, however, remained invariant without response to environmental disturbances; it mainly consisted of Chlorophyta and the Eustigmatophyceae (Stramenopiles).

## Introduction

Algae and Cyanobacteria (collectively referred to as “soil algae” here) form widespread, abundant microscopic photosynthetic communities of microorganisms in the top few centimeters of the soil profile. The diversity of soil algae is immense, as evidenced by morphology-based compilations of cultivable or directly observed species (Ettl and Gärtner, 2014; Metting, 1981). In the upper soil layer, these organisms are most exposed to environmental and seasonal changes. Soil is the largest repository of organic matter on land. The amount of soil-stored C is about the same as in the aboveground vegetation and atmosphere combined (Crowther et al., 2019). The magnitude of the soil organic C pool strongly depends on microorganisms (Jassey et al., 2022; Liang et al., 2019). Modern research on soil microorganisms primarily focuses on heterotrophic microbes and their role in C release, with less attention paid to microbial photosynthesis (Jassey et al., 2022; Liang et al., 2019). Algae and Cyanobacteria are crucial for photoautotrophic input of energy and C (as well as O_2_) into young and developing soils. Cyanobacteria also provide nitrogen fertilization to the soil through their ability to fix nitrogen from the atmosphere.

Globally, soil algae are responsible for about 6% of the net primary production of terrestrial vegetation (Jassey et al., 2022). In arable land, soil algae can constitute up to 27% of the microbial biomass (Abinandan et al., 2019). As photoautotrophic microorganisms, soil algae are among the first colonizers of bare soil and play a key role in soil formation (Pepper et al., 2015). Increased carbon input from the photoautotrophic soil algae can stimulate other microbes’ and also invertebrates’ activities and improve nutrient cycling (Alvarez et al., 2021). Because many cyanobacteria can fix atmospheric nitrogen, they are investigated to be utilized as potential renewable fertilizers for agriculture (Alvarez et al., 2021; Ramakrishnan et al., 2023). Soil algae are essential for maintaining healthy, fertile soil. The cell walls of eukaryotic cells act as reservoirs, absorbing nitrates, phosphates, and certain metals, then gradually releasing them back into the environment, thereby enhancing the soil’s capacity to retain these substances (Alvarez et al., 2021). The gelatinous extracellular polysaccharides produced by soil algae influence small-scale hydrological conditions and promote the growth of other microorganisms. Soil algae produce a diverse array of substances, including pigments, cell wall polysaccharides, mucilage, lipids, and other bioactive compounds (Metting, 1981, 1996; Starks et al., 1981), making them important resources for phagocytic protists in the soil food web (Seppey et al., 2017). Therefore, soil algae have numerous valuable effects on the soil and support diverse ecological functions. Some species of soil algae may even have the potential for biocontrol of plant pathogens or to promote plant growth in agriculture (Alvarez et al., 2021; Ramakrishnan et al., 2023).

Despite their ecological importance, soil algae in arable land topsoils remain poorly studied (Joseph and Ray, 2024). To our knowledge, there are very few studies, if any, focusing on soil algae of arable land in Central Europe. The factors determining their biodiversity are still unknown. In previous studies of soil algae, predominantly a morphospecies approach has been employed, involving direct cell counting or isolating microalgae under laboratory conditions and comparing their microscopic features. This approach is time-consuming, requires experienced scientists in taxonomy and ecology, and may still underestimate actual biodiversity (Huo et al., 2020). Therefore, for assessing the biodiversity of soil algae, a culture-independent approach, i.e., DNA metabarcoding, is preferred over direct cell counting and culture-based approaches, which most previous studies have used (Lin et al., 2013; Lukesova, 1993; Zancan et al., 2006). DNA metabarcoding, as a culture-independent method, has already been employed to arable soil algae in a few studies, but these studies remained at class-level identification (Lentendu et al., 2014; Zhang and Lv, 2022). Additionally, these studies collected the top 10 cm of soil, which probably led to an underestimation of the soil algae abundances; soil algae may be most abundant in the first two centimeters of the topsoil (Lukesova, 1993).

The surface soils of arable lands—due to the constant changes and disturbances caused by agricultural practices—create a challenging environment that the sensitive soil algae and Cyanobacteria must respond to. The intensification of agriculture remains a primary threat to global biodiversity (Jaureguiberry et al., 2022; Tsiafouli et al., 2015); it has already decreased the diversity of arable plants (Fried et al., 2010; Meyer et al., 2013). In this context, the organic management system has been found to support greater biodiversity (Gayer et al., 2021). Agriculture-based factors such as crop choice, sowing density, and light availability are of great importance for plant species diversity. Similarly, different agricultural management systems can host distinct microbial communities (Hartmann et al., 2015). Agricultural land-use intensity, in particular tillage and the use of chemical crop protection, likely also affects the species composition and abundance of soil algae communities (Zancan et al., 2006). Additionally, soil algal distribution in arable fields may depend on the cultivated crop and on multiple abiotic soil parameters, including pH, available organic matter, and nitrogen (Zhang and Lv, 2022). A study comparing soil algal communities of Chinese arable lands found a higher algal diversity on fields planted with *Panicum miliaceum* than on those with *Zea mays* production (Zhang and Lv, 2022). For other soil habitats, an impact of vegetation, season, pH, and nitrogen on the soil algae communities has been suggested (Kostikov et al., 2001; Lin et al., 2013; Maltsev et al., 2017; Zancan et al., 2006). A study of abandoned fields found higher soil algal abundances in autumn and spring than in summer (Lukesova, 1993). In contrast, a study with the focus on bacterial diversity in arable land, found the highest abundances for Cyanobacteria in summer and autumn (Lauber et al., 2013). Soil algal communities might even be connected to vegetation phenology. For arable plants, it is known that the diversity is higher at the field margins than in the interiors (margin effect; Gayer et al., 2021; Marshall, 2004; Rotchés-Ribalta et al., 2015; Seifert et al., 2015, 2014). Whether there is a margin effect also for soil algae remains unknown.

Our study aims to test two main hypotheses, i.e., (1) Different crop phenologies across the year offer constant changes in open-soil availability at different air temperatures, challenging pioneer colonization by soil algae. In response to such disturbances, the composition of the soil algal communities changes. (2) Organic versus conventional farming introduces additional disturbances in the arable field topsoils. The absence of mineral fertilizers and chemical pesticides in organic farming promotes a higher soil algal biodiversity than conventional farming. Organic fertilization, compared to conventional treatment, may increase the abundance of microalgae (Lentendu et al., 2014). Agricultural crop phenology produces major seasonal changes that alter the associated microclimate (Leuschner and Ellenberg, 2017; Seifert et al., 2014). Our study focuses on the surface soils of exemplar arable land where winter wheat (*Triticum aestivum*, Poaceae) is produced under the organic management system. It is usually cultivated as a winter annual of arable lands in Central Europe. It is sown in autumn (usually September-October), overwinters, and is harvested from July onwards. It requires a period of cold (vernalization) to flower. It follows that the first time point of the year, March, presents an open stage with high bare-soil availability on the fields’ soil surfaces, with wheat seedlings still small after the cold period. In July, bare-soil availability is minimal due to the nearly closed crop canopy shortly before harvest. It followed a period of increased air temperatures. Then, in November, shortly after harvest and sowing of the new crop, while still before the period of cold, bare-soil availability and air temperatures are comparable to those in March, but soil rest was only a short period as air temperatures declined. The organic management system (organic farming) enhances total microbial abundance and activity in agricultural soils globally (Lori et al., 2017; Sanders et al., 2025). The avoidance of synthetic chemical pesticides and mineral nitrogen fertilizers, together with the application of diverse crop rotations with intercropping, constitute key farming practices that affect soil microbial community size and activity (Lori et al., 2017). As a first test for possible effects of contrasting farming systems on the diversity of the soil algae, we included arable fields under the conventional farming. The latter fields had wheat or corn (*Zea mays*, Poaceae) as crop plants and were geographically neighboring to minimize differences in soil parameters. Samples were taken at the July time point, when both crops were at their densest growth stage, just prior to harvest. In addition, our study included testing for possible correlations between diversity changes in the soil algal communities and physicochemical soil parameters. To test for a possible margin effect, which is known for the field-margin vegetation (Sanders et al., 2025), topsoil samples were compared from the field interiors and corresponding margins. Finally, to optimize accuracy in the genotypic comparisons, multiple (five) DNA barcode markers were employed.

## Materials and methods

### Study area and sampling

The examined arable fields with wheat as crop under organic farming were located within the Hessian State Domain Frankenhausen (Athmann and Möller, 2025), near the city of Kassel, Germany (Supplementary Figure S1 and Supplementary Table S1, sheet 2). At the time of sampling (2024), the five studied fields (F1–F5) had been managed organically for more than 25 years. The soils ranged from clayey silt (fields F1, and F2) to clayey silt/silty clay (fields F3, F4, and F5). Surface soil from the top three centimeters was collected using a sterilized teaspoon. Fresh samples were transported on ice to the laboratory and kept frozen at −20°C in a freezer for 48 h before being used in the laboratory. Samples were collected from the five same fields on March 1st (samples with IDs FF1–FF5), July 3rd, (samples with IDs FS1–FS5), and November 1st (samples with IDs FH1–FH5) of 2024 (Supplementary Figure S1 and Supplementary Table S1, sheet 2). Additionally, five fields under conventional farming adjacent to those of the Hessian State Domain Frankenhausen (FK1–FK5; Supplementary Figure S1) were sampled at the July sampling point for comparison (sample IDs with FK1–FK5; Supplementary Table S1, sheet 2). The comparison fields had wheat (fields FK3–FK5) or corn (fields FK1 and FK2) as crop plants. Monthly averages of air temperatures and precipitation for the year 2024 in the federal state Hesse, Germany, were obtained from the *Deutscher Wetterdienst* (weather and climate service for Germany; available at: opendata.dwd.de, accessed 12 February 2026). Soil samples were collected along two transects. One transect was placed parallel to the field margin (0.25 m into the field; sample IDs with “A”), and the other was placed 15 m from the margin transect into the field (sample IDs with “I”; Supplementary Table S1, sheet 2). Both transects were 50 m long and about 1.5 m wide. Along each transect, five subsamples were collected at 12.5 m intervals and pooled.

### DNA extraction, PCR, and sequencing

Total DNA was extracted with the DNeasy PowerSoil Pro Kit (Qiagen) in triplicate, following the manufacturer’s instructions. DNA concentration was measured using the Qubit3 Fluorometer (Invitrogen) with the Qubit dsDNA HS Assay Kit (Life Technologies, Carlsbad, CA, United States), following the manufacturer’s instructions. Purified DNA was subjected to PCR with multiple primers targeting plastid regions of the 23S rDNA (UPA, Sherwood and Presting, 2007) and the *rbc*L gene (primer combinations GA*rbc*L for Chlorophyta and Streptophyta, and XD*rbc*L for stramenopile algae; Supplementary Table S1, sheet 3), as well as the nuclear-encoded regions 18S V4 (Stoeck et al., 2010) and ITS2 (Toju et al., 2012; White et al., 1990). The PCR products were multiplexed with seven-nucleotide-long indices (Kircher et al., 2012; Supplementary Table S1, sheet 3). PCR reactions (30–35 cycles) were performed for all three DNA replicates using the Phusion High-Fidelity DNA Polymerase (Thermo Fisher Scientific, United States) according to the manufacturer’s instructions and under conditions previously described (for details, see Supplementary Table S1, sheet 3). For the newly designed primer pairs, GA*rbc*L and XD*rbc*L, the annealing temperatures were 60 and 55°C, respectively. PCR products were purified on a 96-well plate using MagSi-NGSPREP Plus (Magtivio, Netherlands) according to the manufacturer’s instructions. All purified PCR products were pooled, with a maximum of 40 amplicons per pool. DNA was precipitated with ethanol (Zeugin and Hartley, 1985), diluted with sterile HPLC-grade water, and the DNA concentration was measured with the Qubit dsDNA HS Assay Kit as above. The DNA concentration was adjusted with water to the appropriate level for sequencing. Illumina paired-end sequencing was performed at Novogene GmbH (Martinsried, Germany) on a Novaseq6000 platform with an SP flow cell and v1.5 (500 cycles) chemistry (2 × 250 paired-end reads).

To improve taxonomic resolution, many algae strains were sequenced for their 23S UPA regions, i.e., 116 Xanthophyceae, nine Eustigmatophyceae, and one Chlorophyceae, and the *rbc*L gene sequences of eight strains of Xanthophyceae (Supplementary Table S1, sheet 4). Form those algal strains, DNA was extracted from laboratory cultures using the Invisorb^®^ Spin Plant Mini Kit (Invitek) according to the manufacturer’s instructions. DNA concentration was measured as described above. From the strains, the 23S UPA marker was amplified as described previously (Sherwood and Presting, 2007). From the strains of Xanthophyceae, *rbc*L gene sequences were amplified as previously described (Rybalka et al., 2020). For PCR amplification, the MyTag Polymerase (Bioline, Meridian Bioscience, Cincinnati, OH, United States) was used following the manufacturer’s instructions. Amplicons were sequenced using the Sanger sequencing services of Microsynth Seqlab GmbH (Göttingen, Germany). Sequence assembly was performed with the R package *sangeranalyseR* (Chao et al., 2021) or with the DNA-Dragon DNA sequence contig assembler (SequentiX—Digital DNA Processing, Klein Raden, Germany; available at: www.dna-dragon.com, accessed 13 February 2026).

### Sequence processing for metabarcoding

All analyses were performed in R version “4.3.3” (R Core Team, 2024) using RStudio (Posit Team, 2024). Raw sequences were demultiplexed, and adapters and primers were trimmed using cutadapt v3. 3 (Martin, 2011). The *e*-value was set to 0.05, and the overlap value was set to 25 to minimize the effect of cross-contamination. Sequences were further processed, and amplicon sequence variants (ASVs) were identified using the *DADA2* workflow (Callahan et al., 2016). Taxonomy was assigned to the ASVs using *BLAST* + (Altschul et al., 1990; Camacho et al., 2009) against the whole GenBank Nucleotide database (Benson et al., 2018; NCBI GenBank Flat File Release 260, April 2024). The *taxonomizer* R package^1^ was used to assign taxonomy. The names of eukaryotic taxonomic groups were manually adjusted to follow recent taxonomies (Adl et al., 2019; Büdel et al., 2024). The resulting ASV table was filtered and sorted using our own R script. For filtering, all ASVs not assigned to algae groups as defined in Büdel et al., 2024 were removed. Then, rarefaction was performed using the remaining soil algal ASVs by implementing the R packages *phyloseq* and *mirlyn* (Cameron et al., 2021; McMurdie and Holmes, 2013; Schloss, 2024). Next, all ASVs with an abundance < 0.1% were set to zero to reduce the effect of false positives. The remaining ASVs were used for algae identification and to calculate phylogenetic group abundances. Additionally, for the analysis of the microeukaryotic community, the ASV table from the 18S V4 dataset without prior filtering to include non-algae ASVs was processed (rarefaction and 0.97 threshold). To facilitate comparison between ASVs and putative reference sequences, we normalized each bit score S’ with respect to the corresponding query sequence length (Rybalka et al., 2023). The normalized bit score, referred to as *NB*, was maximal (*NB* = 1. 85) using the BLASTN command in the *BLAST* + software. NMDS (non-metric multidimensional scaling), dbRDA (distance- based redundancy analysis), PERMANOVA, and ANOSIM were performed using the R package *vegan* (Oksanen et al., 2013). For heatmaps, the *phyloseq* R package (McMurdie and Holmes, 2013) was used, and for the boxplots the R package *ggplot 2* (Wickham, 2016). Diversity indices were calculated using the *richness*() function in *phyloseq* (McMurdie and Holmes, 2013). Barplots were visualized using LibreOffice (Version: 25.2.5.2.2.5.2, The Document Foundation) and Inkscape (Version 1.3.1.3.2, Inkscape Developers).

The phylogenetic diversity of the *rbc*L ASVs assigned to Xanthophyceae (Supplementary Table S1, sheet 15) were estimated using phylogenetic placement. Alignments of reference and ASV sequences assigned to Xanthophyceae were performed in MAFFT version 7 (Katoh et al., 2019) and the alignment subjected to IQTree2 (Minh et al., 2020; Nguyen et al., 2015) for the construction of Maximum Likelihood (ML) trees (Supplementary file S3). The final trees were visualized and graphically processed in iTOL (Letunic and Bork, 2024) with final editing in Inkscape (Version 1.3.2, Inkscape Developers).

### Soil physicochemical properties and chlorophyll content

For C and N measurements, soil was dried at 60°C for 48 h, sieved (2 mm), and placed in a desiccator to allow residual water to evaporate. Dry samples were analyzed on a Vario Max (Elementar, UK Limited) according to the manufacturer’s instructions. In parallel, another soil sample was dried at 40°C for 48 h, and pH was measured in 0.01 M CaCl_2_ (Merck, Darmstadt) solution. Electrical conductivity (EC) was measured in a soil:water (1:5) solution (Nilo et al., 2021). Chlorophyll was extracted with DMSO as described previously (Caesar et al., 2017), and chlorophylls *a* and *b* were measured with an Agilent BioTek Epoch Microplate Spectrophotometer (Agilent Technologies, Santa Clara, California, United States). For soil moisture, fresh soil was weighed, dried at 105°C for 48 h, and weighed again.

## Results and discussion

### Community composition and variation across time points

**FIGURE 1:**
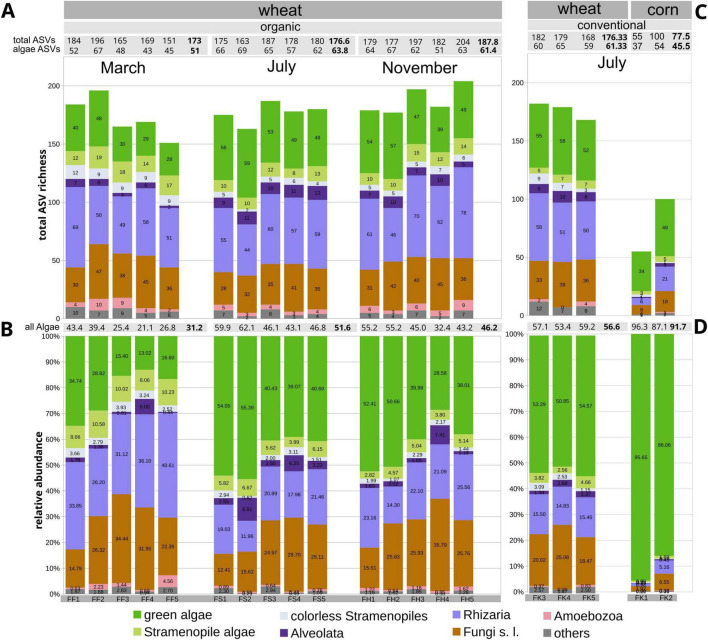
Barplot showing the dynamics of the microeukaryotic community structure in the studied arable fields, based on amplicon-based metabarcoding using the 18S marker (unfiltered). **(A,B)** Variation across the three time points (March, July, and November) as determined by the phenology of wheat (*Triticum aestivum*) as the crop under organic farming. **(A)** Total ASV counts (richness). **(B)** Relative abundances based on read counts. **(C,D)** Comparison between wheat and corn (*Zea mays*) as the crop plants under the conventional farming system at the July time point.

Metabarcoding analyses using the 18S marker revealed that the eukaryotic soil algae comprised of the Stramenopiles (Xanthophyceae, Diatomeae, and Eustigmatophyceae), the Chlorophyta (Chlorophyceae, Trebouxiophyceae, and Ulvophyceae), and the Streptophyta (Klebsormidiophyceae). In addition, few ASVs assigned to Dinophyceae (Alveolates) were found. With the 18S marker, a total of 193 ASVs could be attributed to algae across all samples (*n* = 20; Figures 1A,B and Supplementary Table S1, sheet 5). That is, the soil algae made up 27.4% of the 704 microeukaryote ASVs detected across all samples (*n* = 20) from the studied arable field topsoils. The ASVs of eukaryotic algae made up about one half (42 ASVs) among the top 100 ASVs of all microeukaryotes (Supplementary File S2, page 5), demonstrating their wide abundance among all microeukaryotes in the arable land surface soils. The 18S marker generally amplifies all eukaryotes without targeting a specific group. The stramenopile algae included members of the Xanthophyceae (20 ASVs), Diatomeae (34 ASVs), Eustigmatophyceae (1 ASV), apart from putatively colorless representatives of Chrysophyceae (11 ASVs; Supplementary Table S1, sheet 5). The green algae comprised members of Chlorophyta, i.e., the classes Chlorophyceae (90 ASVs), Trebouxiophyceae (35 ASVs), and Ulvophyceae (nine ASVs), and members of the Streptophyta, i.e., Klebsormidiophyceae (two ASVs; Supplementary Table S1, sheet 5). The Dinophyceae encompassed two ASVs with moderate sequence similarity (*NB* ≤ 1.73) to “unidentified Heterocapsaceae” environmental samples from soils; thus, their genus-level identity remained unclear. The single member of Eustigmatophyceae had maximal sequence identity (*NB* = 1.85) with species of *Vischeria*.

**FIGURE 2:**
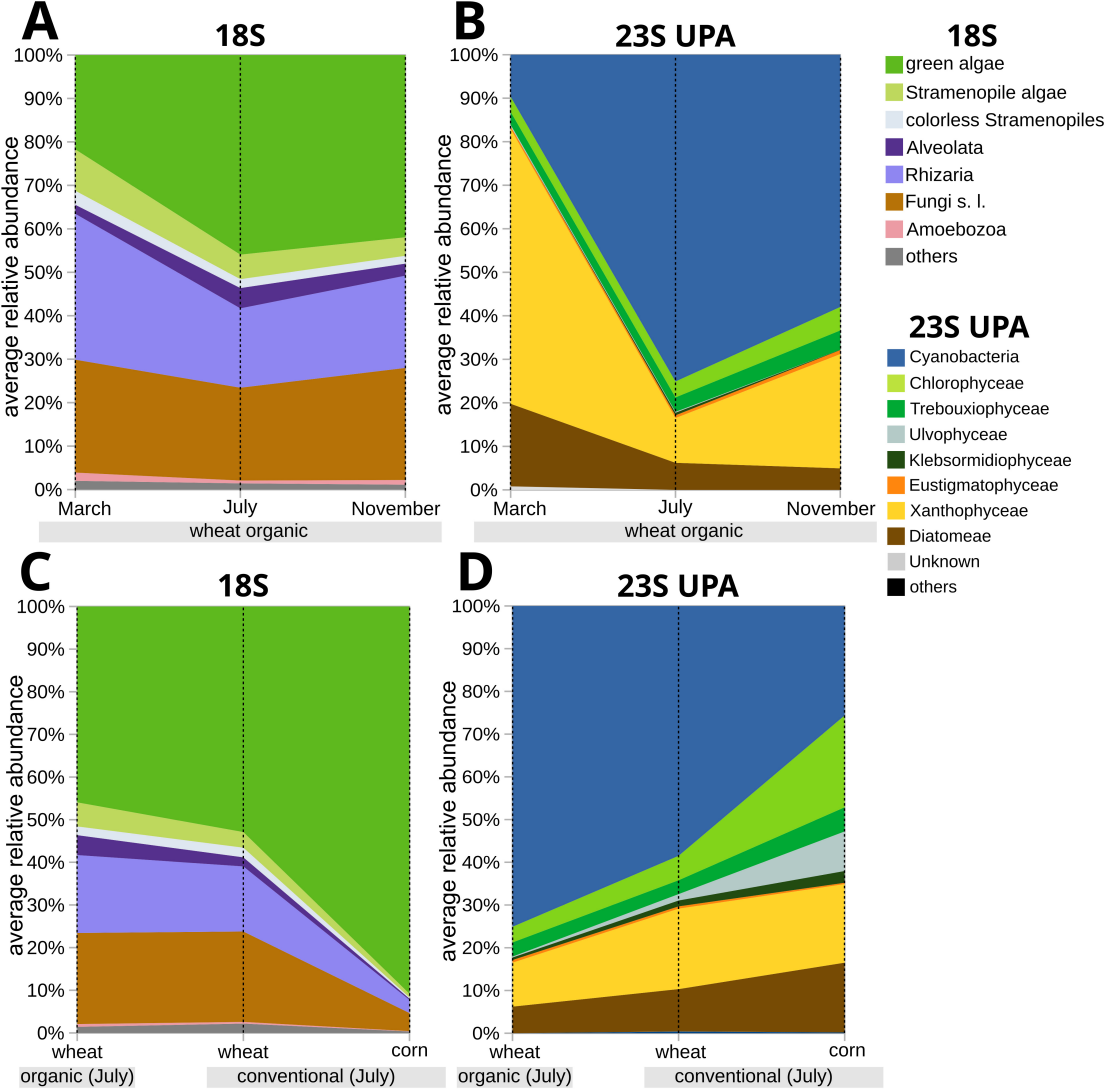
Area graphs illustrating the dynamics of soil algal groups in the surface soils of the studied arable fields across the three time points (March, July, and November), as determined by the phenology of wheat (*Triticum aestivum*) as the crop plant under organic farming **(A,B)**, between the two contrasting farming systems **(C,D)**, and between wheat and corn (*Zea mays*) as the crop plants under the conventional farming system. The boundaries between the areas are based on average relative abundance values. **(A,C)** Based on eukaryotic soil algae only (18S marker), whereas **(B,D)** are based on all soil algal groups, including the Cyanobacteria (23S UPA marker).

In the 18S metabarcoding analysis, the ASV counts of all algae together, averaged 51.0 in March, 63.8 in July, and 61.4 in November without significant (*t*-test, *p* > 0.01) variation across the same fields (Figure 1A and Supplementary Table S1, sheet 7). However, in the relative abundances of all eukaryotic algae based on read counts, there was a significant (*t*-test, *p* < 0.01) increase from March (31.2% on average, *n* = 5) to July (51.6% on average, *n* = 5), followed by a small non-significant decrease in November (46.2 % on average, *n* = 5; Figures 1B, 2A and Supplementary Table S1, sheet 8). It indicated a variation of the algal diversity across the three time points. Based on the 18S marker and ASV numbers, the green algae and the stramenopile algae were the two major groups of soil algae. The green algae significantly dominated in total ASV counts over the stramenopile algae at all three sampling dates using the 18S marker (*t*-test, *p* < 0.01; Figures 1A,B, 2A). At the March time point, the relative abundance of the green algae was lowest with 21.7% on average per sample (*n* = 5), while that of the stramenopile algae was with 9.5% the highest; the difference was, however, not significant (*t*-test, *p* < 0.01). Accordingly, at the March time point, the stramenopile algae reached with 16 ASVs their highest ASV counts on average (*n* = 5). Conversely, at the July time point, the green algae reached their highest relative abundance with 45.9%, whereas the stramenopile algae had their lowest relative abundance with 5.7% (Figure 2A); the differences between both algae groups were significant (*t*-test, *p* < 0.001). At the July time point, the stramenopile algae had with 10.6 ASVs on average (*n* = 5) their lowest ASV counts, and the difference between March and July time points was significant (*t*-test, *p* < 0.01). At the November time point, both algae groups had their lowest abundances, i.e., 41.9% for the green algae, and 4.3% for the stramenopile algae, with the differences between both algae groups being significant (*t*-test, *p* < 0.001; Figures 1B, 2A and Supplementary Table S1, sheet 8).

**FIGURE 3:**
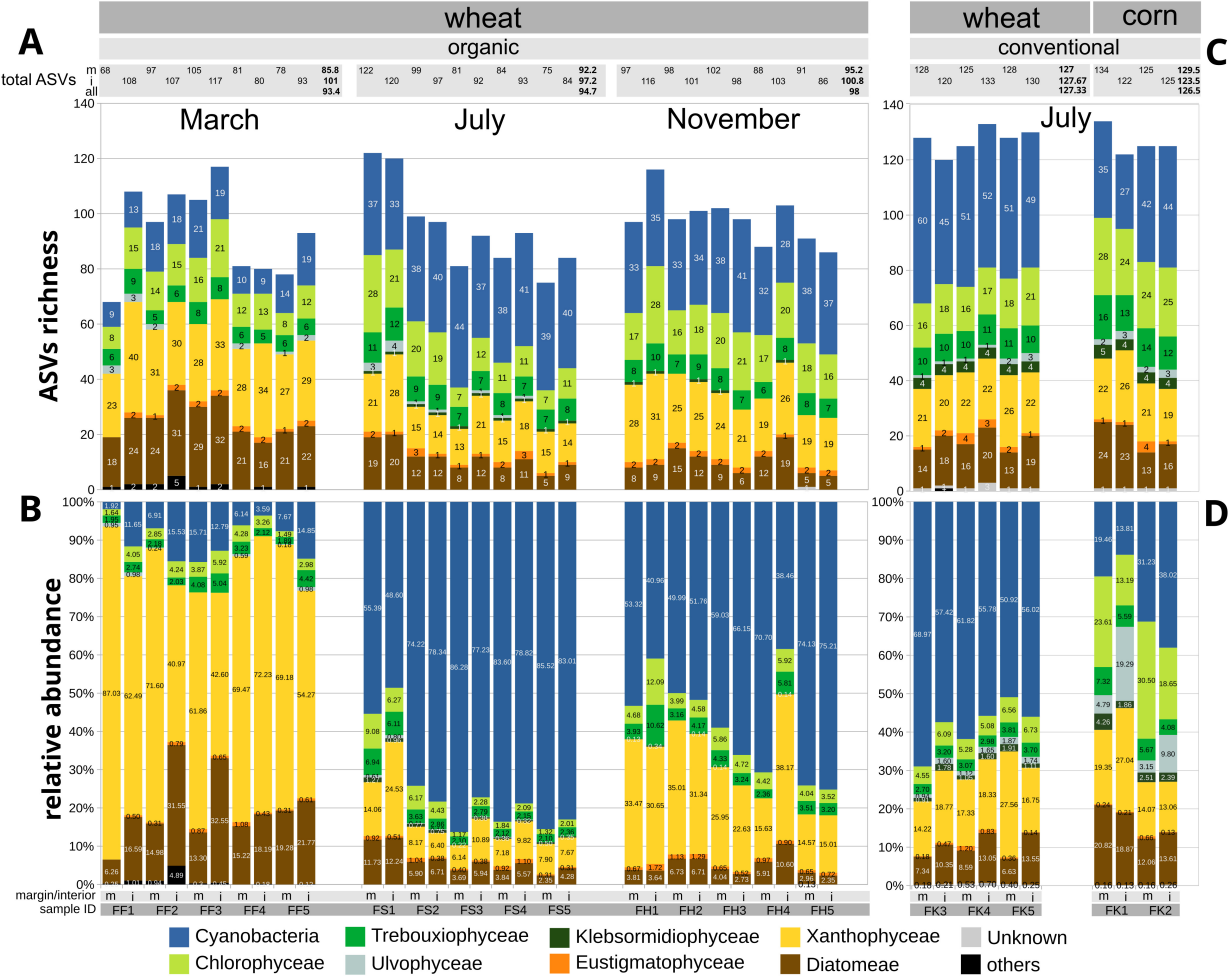
Barplot showing dynamics among the groups of eukaryotic algae and Cyanobacteria in the studied arable fields, based on amplicon-based metabarcoding using the 23S UPA marker. Each field was investigated at its interior (i) and margin (m). **(A,B)** Variation across the three time points, as determined by the phenology of wheat (*Triticum aestivum*) under organic farming. **(A)** Total ASV counts (richness). **(B)** Relative abundances based on read counts. **(C,D)** Comparison between wheat and corn (*Zea mays*) under the conventional farming system at the July time point. Total ASV counts (richness, C) and relative abundances based on read counts **(D)** are shown.

**FIGURE 4:**
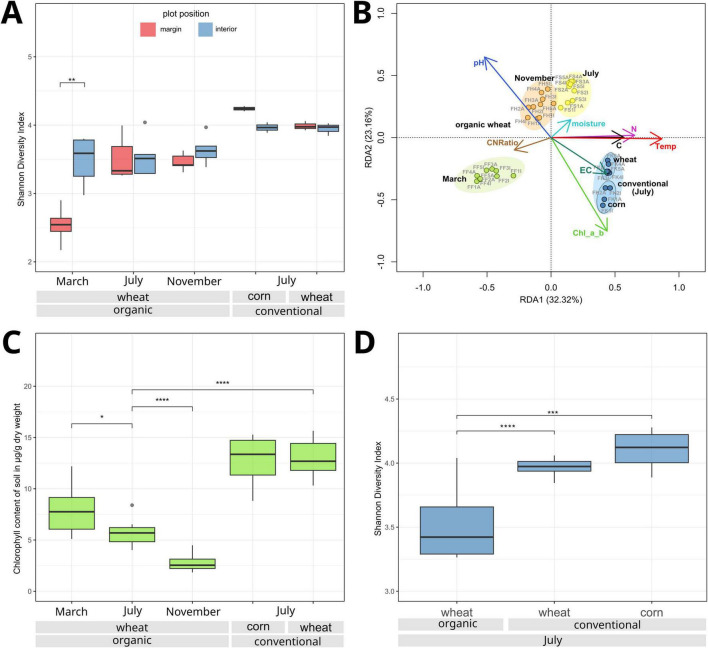
**(A)** Shannon diversity of the groups of eukaryotic algae and Cyanobacteria in the studied arable fields, based on amplicon-based metabarcoding using the 23S UPA marker. Samples from the field interior and margin are compared. **(B)** Redundancy analysis of the photoautotroph communities, based on the 23S UPA marker, calculated with environmental variables. **(C)** Estimation of the algal/cyanobacterial biomass based on the chlorophyll content per gram dry soil weight. **(D)** Comparison of wheat fields under organic and conventional farming systems, and of wheat and corn as crop plants at the July time point. Shannon diversity of the groups of eukaryotic algae and Cyanobacteria, based on amplicon-based metabarcoding using the 23S UPA marker. Significance levels, *p ≤ 0.05; **p ≤ 0.01; ***p ≤ 0.001; ****p ≤ 0.0001.

To include Cyanobacteria and to further test for variation across the three time points, we employed the 23S rDNA gene (UPA) as a marker (Figures 2B,D, 3A,B). It preferentially targets photoautotrophs, i.e., eukaryotic algae and cyanobacteria (Sherwood and Presting, 2007). The analysis included the same samples, i.e., from the field interiors, as used for the 18S rDNA, and, in addition, samples from the margin of the same fields. It made up a total of *n* = 10 samples per time point to test for a potential margin effect (Marshall, 2004). For the eukaryotic algae, the same classes as with the 18S marker were recovered with the 23S UPA marker, but the total number of eukaryotic algae ASVs increased to 269. Of the Cyanobacteria, a total of 124 ASVs was recovered (Supplementary Table S1, sheet 9). In the organically treated wheat fields, the average of all ASVs counts for algae and cyanobacteria per sample (*n* = 10) across the three time points was 93.4, 94.7, and 98.0 ASVs, respectively, with the variation being non-significant (Figure 3A). Accordingly, Shannon diversity indices for the field interiors were also not significantly different across the three sampling dates (Figure 4A and Supplementary Table S1, sheet 2). In contrast to the 18S analyses, the green algae were not dominating over the stramenopile algae (Xanthophyceae, Diatomeae) with the 23S UPA marker. Rather, at the March time point, the stramenopile algae dominated with an average of 55.7 ASVs per sample significantly over the green algae, which had an average of 21.2 ASVs per sample (*n* = 10, *t*-test, *p* < 0.001, Figures 2B, 3A and Supplementary Table S1, sheet 10). At the July time point, both algae groups had similar average total ASV numbers per sample, i.e., 25.4 and 30.6, and the differences were not significant (*t*-test, *p* > 0.01). At the November time point, the stramenopile algae, with an average of 35.6 ASVs, again significantly (*t*-test, *p* < 0.001) dominated over the green algae, which had 27.4 ASVs on average (Supplementary Table S1, sheet 10). Among the stramenopile algae, the Xanthophyceae dominated over the Diatomeae, e.g., with 30.3 over 23.8 ASVs on average per sample in March. The Xanthophyceae dominance was significant at the March (*t*-test, *p* < 0.01) and November (*t*-test, *p* < 0.001) time points, but not at the July time point (Figure 2B). The few ASVs of Eustigmatophyceae (Stramenopiles), with an average 1.9, and a maximum of four ASVs showed no significant variations across the three time points (Supplementary Table S1, sheet 9). Within the Chlorophyta, the Chlorophyceae significantly dominated (*t*-test, *p* < 0.001) over the Trebouxiophyceae, e.g., with 19.1 over 7.8 ASVs on average per sample (*n* = 10) in November. This relationship was significant at the March and November time points, but not significant at the July time point. Similar to the Eustigmatophyceae, members of the class Ulvophyceae (Chlorophyta) remained almost unchanged across the three time points. Members of Klebsormidiophyceae could not be found in all March samples (Supplementary Table S1, sheet 9). In the Cyanobacteria, a significant variation (*t*-test, *p* < 0.001) between the March and July time points was found, i.e., in their ASV counts as well as relative abundances (Figures 2B, 3A,B). With the Cyanobacteria, there was a significant increase (*t*-test, *p* < 0.001) from on average (*n* = 10) from 15 ASVs and 9.7 % relative abundance in March to 38.7 ASVs and 75.1% relative abundance in July. Between July and November, a decrease to 34.9 ASV counts on average (significant; *t*-test, *p* < 0.01) and a decrease to 58% relative abundance (not significant) were observed (Supplementary Table S1, sheets 9, 10, 11).

We conclude that the UPA 23S marker allowed for a more detailed analysis of the variation in the soil algae communities across the three time points. The 23S UPA marker recovered more ASVs of eukaryotic algae than the 18S marker, and most importantly, allowed to include the Cyanobacteria. While the 18S analyses suggested a significant predominance of green algae over stramenopile algae across all time points (Figure 2A), the 23S UPA analyses revealed the opposite, i.e., a significant dominance of stramenopile algae over green algae at the March and November time points (Figure 2B). However, analysis of similarity (ANOSIM) revealed for both markers, 18S and 23S UPA, that the distinction of three groups of soil algae at the three time points is significant (23S UPA: *R* = 0.903, *p* = 0.0001; 18S: *R* = 0.9503, *p* = 0.0001).

In March, the soil algae community in the arable land topsoils had experienced longer periods of low temperatures (Supplementary Figure S2) while bare-soil availability was high. At the March time point, the stramenopile algae (in particular the Xanthophyceae) had their highest ASV counts and relative abundances with both the 18S and the 23S UPA markers (Figures 1, 2A,B, 3). However, the dominance of the stramenopile over the green algae and Cyanobacteria was only seen with the 23S UPA marker. At the July sampling point, there were contrasting conditions at the soil surfaces manifested in constantly increasing temperatures over 3 months (Supplementary Figure S2) and diminishing topsoil availability due to the dense growth of the crop plant. At the July time point, a rather different composition of the soil algae communities was found. The Cyanobacteria were predominant over the other soil algae, evidenced by their significant increase in ASV counts and relative abundances (Figures 2B, 3A,B). It was accompanied by a significant decrease of the stramenopile algae in ASV counts and relative abundances, found with the 23S UPA marker. In the green algae, however, with the same marker, no significant variation was found between the March and July time points. However, the corresponding variation in green algae was found to be significant with the 18S marker, in the absence of Cyanobacteria, which were left undetected. At the November time point, air temperatures were about the same as at the March time point (Supplementary Figure S2). Soil algae thriving in the warmer summer months may still persist due to their tolerance of lower temperatures, making the soil algal communities at the July and November sampling points more similar to each other than those at the March and July time points. Bare-soil availability may have been comparable at both the March and the November time points. However, at the November time point, the topsoils had experienced recent disturbances by tillage followed by the sowing of new crop seeds. Compared with the July time point, at the November time point there was a significant decrease in Cyanobacteria (*t*-test, *p* < 0.01), whereas stramenopile algae increased significantly using the 23S UPA marker (Figures 2B, 3B). The latter increase was also seen with the 18S marker, but it was not significant. For the green algae, however, there was no significant variation in either marker between the July and November time points (Figures 2B, 3A,B).

The 23S UPA ASVs from all the wheat fields samples (*n* = 30) under the organic management were subjected to a distance-based redundancy analysis (db-RDA), which also included various abiotic soil parameters (Figure 4B and Supplementary Table S1, sheet 2). The analyses unveiled three clusters, each corresponding to a certain time point (Figure 4B). The soil algae clusters of July and November were closer to each other than the March and July clusters. It illustrates the view that at the November time point still many algae ASVs from the warmer summer months can be found. A PERMANOVA test showed that the measured soil parameters could explain 60 % of the variation observed between the samples (Supplementary Table S1, sheet 21). The parameters pH, N, chlorophyll-ab (Chl *a,b*), and temperature were the significant (*p* = 0.001) parameters, and together explained about 40% of the total variation. The strongest positive correlation with the soil parameters was observed between C and N, and between N and EC, while the strongest negative correlation was between Chl *a,b*, and pH (Supplementary Table S1, sheet 20). The July samples were positively associated with temperature, C, and N, whereas the March samples were negatively associated with these parameters (Figure 4B). The Chl *a,b* content was used as a proxy for algal biomass in the surface soils. Significant differences in algal biomass were observed across the three time points (Figure 4C).

We conclude that there is a clear dynamic in the soil algae communities of arable fields, seen in the variation across the three time points in March, July, and November, with wheat as a crop and under an organic management system. It is evidenced by significant shifts among the soil algae groups as well as in their biomass. Interestingly, among the stramenopile algae there was a significant (*t*-test, *p* < 0.01) increase in ASV counts and relative abundances between the July and November time points only in the Xanthophyceae, but not in the Diatomeae (Figures 2B, 3 and Supplementary Table 1, sheets 10, 11). Unlike Xanthophyceae, which seemed to have responded to the changing environment fast (July to November), for the Diatomeae, the time after crop harvesting may still have been too short to recover similar abundances and species richness as in March. These observations support the view of Xanthophyceae as pioneer microorganisms capable of rapid adaptation to changing environmental conditions (Rybalka et al., 2020). For Diatomeae in soils, potential indicator properties have often been considered (e.g., Vacht et al., 2014; Zhang et al., 2020; and citations therein). However, there is still limited information on the variables affecting the structure of soil diatom communities, unlike aquatic diatoms, for which various diatom-based indices exist to characterize the ecological status of water bodies (Antonelli et al., 2017). For Diatomeae in arable field soil surfaces, direct microscopy of cleaned and sieved soil samples revealed a peak in species richness in the late summer (Antonelli et al., 2017; Berard et al., 2004; Stanek-Tarkowska et al., 2015; Vacht et al., 2014). In contrast, our study observed the highest Diatomeae richness in March (Figure 2B and Supplementary Table 1, sheets 10, 11). This may be explained by the very different methodologies applied; many diatom species may have escaped the previous microscopic analyses. That the Cyanobacteria abundance nearly doubled from March to July, may reflect the significant temporal variation observed in bacterial communities of agricultural field soils (Lauber et al., 2013). It aligns with the positive correlation between Cyanobacteria diversity and temperature observed in a microscopy-based study of arable fields in Southern Iraq’s Middle Euphrates region (Alghanmi and Jawad, 2019). In July, the dense crop cover shading the soil surface might have promoted Cyanobacteria diversity and abundance compared to eukaryotic algae, as terrestrial Cyanobacteria are often found in low-light environments like caves (Albertano, 2012; Popović et al., 2020).

### Margin effect

**FIGURE 5:**
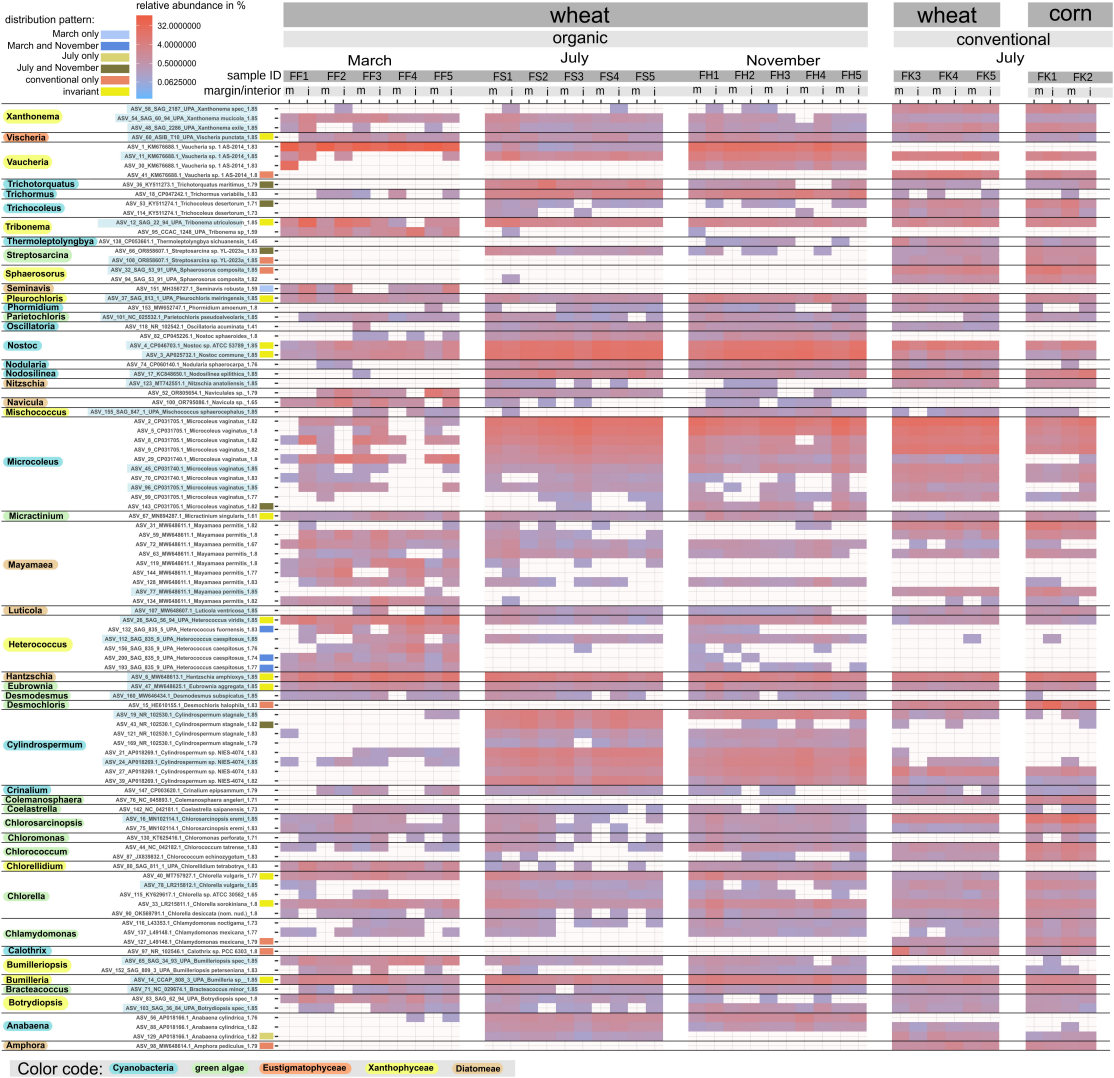
Distribution of the algal and cyanobacterial genotypes (ASVs) across the three time points, the contrasting farming management systems, and the two crop plants, as based on the 23S UPA marker. The six identified distributional patterns (see text and Supplementary File S2) are indicated next to the ASV IDs. Relative abundances (read counts) of the top 100 ASVs of all algal and cyanobacterial genotypes (ASVs; see Supplementary Table S1, sheet 9) are visualized as heatmaps. Each ASV ID is associated with the accession number of its closest reference sequence, the species identification of the reference, and the normalized bitscore (*NB*) of pairing significance to its closest reference sequence. The various taxonomic groups to which the ASVs were assigned are color-coded.

Field margins may serve as refuges supporting higher arable plant diversity than in the field interiors (Gayer et al., 2021; Marshall, 2004; Marshall and Arnold, 1995; Seifert et al., 2015). However, for soil algae, we found only a difference in the abundance of a single predominant ASV with the 23S UPA marker. At the March time point, the ASV_1 *Vaucheria* sp. (Xanthophyceae) accounted on average (*n* = 5) for 53% of all read counts at the margins of all five organically treated wheat fields. In the field interiors, the same ASV accounted for only 21.5% of the read counts, a difference that was significant (*t*-test, *p* < 0.01) (Supplementary Table S1, sheet 9). This corresponded to the field observation of a mass development on the soil surface in those fields at the March time point, as also indicated by very high read counts of the *Vaucheria* ASVs (Figure 5 and Supplementary File S2). Apart from ASV_1, however, no other significant differences were found between the field margins and interiors of the same five fields, either at the March time point or at the other time points (Supplementary Table S1, sheets 9, 10, 11). Consequently, using the Shannon diversity index, there was no significant difference between the interiors and margins of the five fields except at the March time point (Figure 4A). The high abundance of ASV_1 *Vaucheria* sp. may have influenced the analysis, because the Shannon diversity index also incorporates species abundance (Kim et al., 2017). Compared to the margin, ASV counts were slightly higher in the field interiors, but the difference was not significant (*t*-test, *p* > 0.01). This may suggest that greater disturbances from intensive management, as well as higher crop sowing density in the arable field interior, have positive effects on the diversity of surface soil algae. Our analyses were not sufficiently conclusive to determine whether the various environmental factors that differentiate margin and interior habitats of arable fields can affect soil algal diversity. Further tests using a larger number of fields and various crop plants will be required. A margin effect was found for fungi and bacteria with a larger sample size (Aguiar et al., 2023). Other than crop vegetation, i.e., hedgegrows or flower strips in the uncropped field margin may also alter fungal and bacterial diversity and community structure, and effects may depend on phylogenetic groups (Bednar et al., 2023; Ricono et al., 2022). These factors should be considered in future studies investigating the margin effect in the soil algal diversity.

### Management system influence

We compared the soil algal community composition of five organically treated wheat fields with that of three wheat fields under conventional management, which likely included synthetic fertilizers and chemical crop protection. To minimize other variables, we selected the three conventionally treated fields to be directly adjacent to the organically treated fields (Supplementary Figure S1). The comparison was conducted when the crop in both types of fields was at full growth, i.e., at the July time point. With the 18S marker, the comparisons revealed no other significant changes apart from a significant decrease (*t*-test, *p* < 0.01) in the ASV counts of stramenopile algae in the fields under the conventional management system (Supplementary Table S1, sheet 7). Based on read counts-based relative abundances, however, there was no significant variation in stramenopile algae with the 18S marker (Figure 2C). In contrast, the 23S UPA marker revealed a significant increase (*t*-test, *p* < 0.01) in stramenopile algae, in both ASV counts and read counts-based relative abundances, for the conventionally treated fields (Figure 2D and Supplementary Table S1, sheets 10, 11). Within the stramenopile algae, the Diatomeae significantly (*t*-test, *p* < 0.01) increased in ASV counts but not in relative abundances (Figure 2D). Conversely, the Xanthophyceae increased significantly (*t*-test, *p* < 0.01) in relative abundances, but their increase in ASV counts was not significant (Supplementary Table S1, sheets 10, 11). Other significant (*t*-test, *p* < 0.01) increases were observed in the green algal classes Ulvophyceae and Klebsormidiophyceae (relative abundances only). The Cyanobacteria, however, showed a significant decrease (*t*-test, *p* < 0.01) in both ASV counts and relative abundances (Figures 2D, 3).

Soil algal biomass, as estimated from Chl *a*,*b* content, was significantly higher in wheat fields under conventional management than in those under organic management (Figure 4C). The distance-based redundancy analysis (db-RDA) of abiotic soil parameters clearly distinguished the wheat fields under different management systems from each other (Figure 4B). Similar to soil samples from wheat fields under organic farming, those from conventional farming were positively associated with temperature, C, and N (Figure 4B). However, samples from conventionally managed fields also showed positive associations with EC and Chl *a,b*, and a negative association with pH.

We conclude that the conventional management system significantly affected the soil algae communities. First, there was a significant increase in the algal biomass in the surface soils. Second, the conventional management system had a positive effect on the diversity of the stramenopile algae (Xanthophyceae and Diatomeae). The green algae, apart from the Ulvophyceae and Klebsormidiophyceae, remained almost unaffected. The latter two had, however, just very few ASVs. Finally, the Cyanobacteria were the only group that was negatively affected.

### Crop influence

We compared wheat and corn as two distinct crop species at the July time point, when both were at a comparable stage of full growth. Differences in the corn’s phenology relative to wheat include larger plants with greater spacing, less shading of the soil surface, and increased bare soil availability. However, corn production affects multiple soil factors that are not readily separable. For example, corn field soils showed almost three times higher salt content than wheat field soils, along with increased total nitrogen (TN), total carbon (TC; Supplementary Table S1, sheet 2). Increased salinity and acidity can be attributed to the application of mineral nitrogen fertilizer (Han et al., 2015), which in turn can significantly alter the microbial community (Chen et al., 2016; Degrune et al., 2017; Haj-Amor et al., 2022; Kuntz et al., 2013; Lentendu et al., 2014; Zhang et al., 2021). To minimize other variables, we selected two corn fields, FK1 and FK2, directly adjacent to the wheat fields FK4, FK3, and FK5 (Supplementary Figure S1). The studied corn fields, as well as the wheat fields for comparisons, were under a conventional management system.

There was substantial variation in microeukaryote 18S ASV counts across the two corn fields studied, with 55 and 100 ASVs, exceeding those in the three conventionally treated wheat fields. However, for all eukaryotic algae together, their 18S ASV counts and relative abundances varied only slightly in the same corn fields (Figure 1C). The differences with the samples from the conventionally treated wheat fields were not significant (*t*-test, *p* > 0.01). It suggests that the environmental factors that differentiate the surface soils of the studied corn and wheat fields had a smaller effect on the soil algae than on other microeukaryotes in the surface soil. Within the soil algae, the Cyanobacteria significantly decreased, whereas the green algae significantly increased in their relative abundances using the 23S UPA marker in relation to the conventionally treated wheat fields (*t*-test, *p* < 0.01; Figure 2D and Supplementary Table S1, sheet 11). The corn field samples exhibited an increased soil algal biomass as indicated by higher Chl *a,b* content (Figure 4C), and a slightly higher Shannon diversity index (Figure 4D) in relation to the conventionally treated wheat fields, but the differences were not significant (*t*-test, *p* > 0.01). Therefore, in the db-RDA, the corn and wheat field clusters were distinct from each other, but very close to each other within the cluster of samples from all fields under the conventional management system (Figure 4B).

We conclude that corn, as a crop, compared to wheat, influenced the diversity of Cyanobacteria and Chlorophyceae in the surface soil of the studied agricultural fields. Green algae were found in low abundances in cropland (Aslani et al., 2024). However, in a study focusing on corn fields, the green algae reached approximately 80% relative abundance of all soil microeukaryotes protists (Li et al., 2024). In contrast to our results (Figure 4D), another study comparing the eukaryotic algae diversity of arable land planted with various crops, found corn fields to exhibit the lowest algae diversity (Zhang and Lv, 2022). Further, wheat fields were shown to support a higher bacterial biomass and enzyme activity than maize (corn) fields (Kramer et al., 2013), emphasizing the effect of selected crops on microbial communities.

### Genotype distribution patterns

We further analyzed the distribution of soil algal genotypes using multiple markers across the studied fields, i.e., five fields across three time points under organic management (30 samples) and five additional fields under conventional management to test for management system and crop influences (10 samples; Figure 5). Genotypes were the ASVs obtained with the markers 18S, 23S UPA, and *rbc*L for the eukaryotic algal groups, i.e., the green algae, Xanthophyceae, and Diatomeae (Supplementary File S2). For the green algae, the ITS2 marker was employed in addition. For the Cyanobacteria, the 23S UPA marker was used (Supplementary File S2). Specifically, we examined the top 50 (Supplementary File S2, all four markers) and top 100 (Figure 5; 23S UPA marker only) most abundant ASVs. Distributional preferences of ASVs were visualized through heat maps that displayed abundances based on read counts (Figure 5 and Supplementary File S2). The displayed abundances of an ASV were treated as either present or absent in a given sample. Using the 23S UPA marker, the top 100 (most abundant) ASVs (Figure 5) were mainly distributed among the Cyanobacteria (35 ASVs), the Xanthophyceae (25 ASVs), the Diatomeae (16 ASVs), Chlorophyceae (13 ASVs), and Trebouxiophyceae (seven ASVs). The Klebsormidiophyceae had only two ASVs out of the top 100, and Eustigmatophyceae and Ulvophyceae each had only a single. Given the limitations of previous microscopy approaches, DNA metabarcoding revealed genotypes in the same genera as previous studies. Both approaches agree on the most common genera in Cyanobacteria (e.g., *Microcoleus*, *Nostoc*), Chlorophyta (e.g., *Chlorella*, *Chlorococcum*, *Chlorosarcinopsis*), Diatomeae (e.g., *Hantzschia*, *Mayamea*, *Pinnularia*), and Xanthophyceae (*Heterococcus*, and *Xanthonema*), as taken from a recent compilation (Barthel et al., 2026) and previous studies (Lin et al., 2013; Lukesova, 1993; Zancan et al., 2006). However, other genera that DNA metabarcoding revealed as most abundant, e.g., *Vaucheria* (Xanthophyceae), were not discussed in the culture-based studies and must have been missed by those approaches due to biases inherent to the culture method. The Ulvophyceae is commonly recognized as a green algal lineage of macroscopic marine algae, with few transitions to subaerial habitats. Our findings, however, align with recent studies indicating an extended diversity of the class yet to be examined in soil habitats (Darienko and Pröschold, 2017; Rybalka et al., 2023).

Linked to crop phenology, two principally distinct distribution patterns were identified. Temperature and bare-soil availability emerged as the most likely factors influencing these patterns. A preference for the March time point, i.e., highest read counts of an AVS at that time point, was well perceived, but almost exclusively among the stramenopile algae. A March preference was rare among green algae. It was not found in the cyanobacteria (Supplementary File S2). It supports Xanthophyceae and Diatomeae, as the pioneering soil algae, well adapted to prolonged periods of low temperatures when bare-soil availability is high as it was the case at the March time point. Many March-preferring genotypes of stramenopile algae were not observed at the later sampling dates (Supplementary File S2). Examples for the March-preference among Xanthophyceae were ASVs from *Heterococcus*, and among Diatomeae, several ASVs from the genus *Mayamaea* (Figure 5). Those were recovered with all three markers (23S UPA, 18S, and *rbc*L; Supplementary File S2). Additional examples were seen only with the 23S UPA marker in *Tribonema* (Xanthophyceae) and *Seminavis* (Diatomeae; Figure 5). The November time point offered a similarly low temperature (Supplementary Figure S2) as well as a soil surface with high-bare soil availability. However, the similar situation had persisted for a much shorter period than at the March time point. Therefore, mostly soil algae genotypes from the preceding warmer periods that tolerate the cooler temperatures are expected then. A prominent example was the genus *Vaucheria* (Xanthophyceae), within which the same genotypes (ASVs) had similarly high abundances (read counts) in March and November. Those had a lower abundance or were not found at the July sampling date (Figure 5 and Supplementary File S2). Similarly, and because lower temperatures are involved, the pattern of highest abundances in March as well November was preferentially found in the Xanthophyceae, e.g., the genotypes (ASVs) from *Bumilleriopsis*, *Botrydiopsis Heterococcus*, *Pleurochloris*, and *Tribonema.* There were also examples from the Diatomeae, the genera *Gomphoneis*, *Mayamaea*, and *Placoneis* (Supplementary File S2). The March-November pattern was rare in green algae and was not observed in Cyanobacteria, like with the March preference.

In contrast to the stramenopile algae, the Cyanobacteria and green algae provided many genotypes at the warmest time point, July, when bare-soil availability was low. These genotypes were, in a smaller portion, confined to July or mostly found at the July and November time points (Supplementary File S2). Consistent with the RDA analysis of the ASVs, in which all July and November samples formed two neighboring clusters, the presence of the July-November distributional pattern underlines that many soil algal genotypes present at the warmest time point also tolerate lower temperatures (Figure 4B). In the green algae, most genotypes found at the July and November time points, were from the Chlorophyceae, such as *Heterochlamydomonas*, *Pseudomuriella*, and *Tetracystis* (Supplementary File S2). Other green algal genotypes with the same pattern were from *Watanabea* (Trebouxiophyceae) and *Streptosarcina* (Klebsormidiophyceae). In the Cyanobacteria, corresponding examples were from *Cylindrospermum*, *Microcoleus*, *Nostoc*, and *Trichocoleus* (Figure 5 and Supplementary File S2). Examples of genotypes confined to July, were from the green algae *Chlorella*, *Leptosira*, and *Stichococcus* in the Trebouxiophyceae, and from the Cyanobacteria *Anabaena* and *Romeria* (Figure 5 and Supplementary File S2).

We conclude that the distribution patterns of soil algal genotypes suggest the soil algae with a preference for low temperatures and high light availability were mostly stramenopile algae (Xanthophyceae, Diatomeae). In contrast, certain green algae may prefer higher air temperatures and lower light availability at low bare-soil availability, due to a dense crop canopy.

Whether soil algal genotypes adapt to or are sensitive to temperature and varying light levels requires further testing through culture experiments. However, published culture experiments with strains of the same genus are sparse. The most abundant *Vaucheria* genotypes, i.e., the UPA ASV_1 and the *rbc*L ASVs 1 and 3, were most abundant in the March and November samples but nearly absent in the July samples (Supplementary File S2). Although no culture experiments with terrestrial *Vaucheria* strains have been published to date, our observation is consistent with findings from aquatic *Vaucheria*. The latter forms dense coverings submerged in the cold water of brooks and rivers, and optimal growth was observed at low temperatures in culture experiments (Schagerl and Kerschbaumer, 2009). Conversely, some genotypes of *Tribonema* and *Xanthonema* had their highest abundances in the July samples but were also frequently recovered in March and November (Supplementary File S2). This pattern may indicate a preference for warmer temperatures, along with tolerance of lower temperatures. Our observation is consistent with psychrotolerance observed in culture tests of *Xanthonema* and *Tribonema* strains (Gao et al., 2023; Wang et al., 2016). The sensitivity of terrestrial Xanthophyceae to soil surface temperature is also indicated by PERMANOVA, which shows a negative correlation between soil Xanthophyceae and temperature (Supplementary Table S1, sheet 19). *Streptosarcina* (Streptophyta, Klebsormidiophyceae), though recovered at all sampling dates, had its highest read counts in the July samples (Supplementary File S2), resulting in a positive correlation of the Klebsormidiophyceae with temperature (Supplementary Table S1, sheet 19). Cultured strains of *Streptosarcina* were found to be adapted to higher temperatures and lower light levels (Pierangelini et al., 2019). Similarly, several Cyanobacteria genotypes had their highest read counts in July (Supplementary File S2). This pattern may also be correlated with surface soil pH. In the studied fields, the pH was slightly higher in the July samples than in those from March (Supplementary Table S1). For *Cylindrospermum* ASVs, a significant positive correlation with pH was observed using the Pearson correlation (Supplementary Table S1, sheets 16, 17, 18). For some soil cyanobacteria, a general preference for an alkaline pH has been reported, although some species can have a wider range (Alghanmi and Jawad, 2019; Lukešová and Hoffmann, 1996; Whitton, 2012). In contrast, several genotypes from the genus *Microcoleus* had the highest read counts in the more acidic topsoils of the conventionally treated fields (Supplementary File S2). A significant negative correlation with pH was found for one particular *Microcoleus* ASV (ASV_70; Supplementary Table S1, sheet 18). A recent study showed that strains of *Microcoleus* can thrive even at pH values as low as 4 (Yifan et al., 2024).

The conventional management system, which involves the use of fertilizers and pesticides, introduced additional soil algae genotypes not found in the organically managed topsoils across the three time points, and independent of wheat or corn as the crop plant. Those genotypes were not recovered from the organically treated fields. They were found across all soil algae, but with the majority found in the green algae. Such a conventional-only pattern was found within the Chlorophyceae with examples from the genera *Chlorosarcinopsis*, *Tetracystis*, and *Protosiphon*. Other green algal examples were in *Deuterostichococcus* (Trebouxiophyceae), *Desmochloris halophila* (Ulvophyceae), and members of the Klebsormidiophyceae, i.e., *Klebsormidium* and *Streptosarcina* (Figure 5 and Supplementary File S2). Fewer examples for the conventional-only pattern were from the stramenopile algae, i.e., the Diatomeae, with examples in *Mayamaea*, and *Stauroneis*, and Xanthophyceae, with examples in *Botrydium* and *Sphaerosorus*. The conventional-only pattern was also found in the Cyanobacteria, e.g., *Calothrix*, *Microcoleus*, and *Phormidesmis* (Figure 5 and Supplementary File S2). That the conventional-only pattern was frequently observed further supports the conclusion that the type of management system in arable fields is a significant determinant of soil algal biodiversity in arable fields. No soil algal genotypes were found that could discriminate between wheat and corn in the conventionally treated fields. For *Desmochloris* and *Sphaerosorus*, the Pearson correlation analysis at the level of ASVs showed a significant positive correlation with salt content (EC) and a significantly negative correlation with pH (Supplementary Table S1, sheet 18). In addition, *Sphaerosorus* was the only member of Xanthophyceae with a positive correlation with temperature. *Klebsormidium* and *Streptosarcina* showed significant positive correlations with salt content (EC), total nitrogen (TN), and temperature, but a significant negative correlation with pH, which explains their preferential occurrence on slightly more acidic soil surfaces under the conventional management system, likely on soil surfaces treated with synthetic fertilizers.

Finally, a large group of soil algae genotypes remained invariant. These genotypes were present at high abundance across all samples, regardless of time point, management system, or crop plant. They showed no response to the various and constant changes or disturbances in their habitats. They may be considered a robust and productively growing permanent stock of soil algae in the topsoils of arable land. The majority of those invariant genotypes were from the green algae (e.g., *Eubrownia*, *Streptosarcina*), with only a few found in the stramenopile algae (e.g., Xanthophyceae*: Heterococcus, Vaucheria*, and Eustigmatophyceae: *Vischeria*) and a single genus in the Cyanobacteria, i.e., *Nostoc* (Figure 4 and Supplementary File S2). Their tolerance of harsh environmental conditions requires investigation through future culture experiments.

### Multiple markers for resolving community composition

Our study used two universal markers, 18S V4 and 23S UPA, to evaluate soil algae diversity across lineages. Generally, NGS metabarcoding to identify microbial taxa is known to be affected by biases that occur during PCR amplification (Lavrinienko et al., 2021). Comparison between these markers showed a marker-specific bias. Both markers revealed contrasting total ASVs counts. For Xanthophyceae (Stramenopiles), the 23S UPA identified 82 ASVs, while the 18S marker found only 36 ASVs (Supplementary Table 1, sheets 6, 9). Conversely, for Chlorophyta, the 23S UPA detected only 88 ASVs, whereas the 18S marker identified 190 ASVs. The 23S UPA primers are designed to amplify all phototrophic algae and cyanobacteria (Sherwood and Presting, 2007). Relying on “universal” amplicons likely results in under-representation of many microbial groups in these surveys (Stoeck et al., 2010). Prior studies comparing various markers and primer sets that focused on green algae concluded that 23S UPA might not be recommended for evaluating green algae diversity because it may lack sufficient variability to distinguish among species (Hall et al., 2010; Saunders and Kucera, 2010). These studies also argued that introns in the 23S UPA region in many green algae could compromise the marker’s reliability for these groups. With 18S V4, also the study of Stoeck et al. (2010) found considerably fewer Stramenopile algae compared to green algae. Another study found that Eustigmatophyceae (Stramenopiles) were not amplified with the same 18S V4 primers, despite these algae being observed by light microscopy in phytoplankton samples (Salmaso et al., 2022). Amplicon length may also influence detection, with some Xanthophyceae taxa potentially escaping sequencing detection (Rybalka et al., 2023). Unlike green algae, where the amplicon length of the 18S V4 region was nearly consistent (377–381 nts), Xanthophyceae showed notable length variation (377–410 nts; Supplementary Table 1, sheets 6, 9). This suggests that even longer 18S V4 regions may exist within Xanthophyceae. These, as well as those with group I introns within the 18S V4 region, could have escaped our sequencing approach. Compared to the universal markers, lineage-specific amplification increased the total ASV counts for Xanthophyceae and Chlorophyta. The primer set XD*rbc*L yielded 107 Xanthophyceae ASVs, even more than with the 23S UPA marker (Supplementary Table 1, sheet 15).

To optimize the reliability of the amplicon-based metabarcoding approach for resolving the composition of the soil algal communities, multiple markers were used. In amplicon sequencing approaches for eukaryotic algae, multiple primer sets have been regarded advantageous for gaining a more complete understanding of community structure (Bradley et al., 2016). When regarding ASVs with high identities wtih their closest references (*NB ≥* 1.75; Table 1), the nuclear markers ITS2 and 18S V4 markers yielded the best recovery with highest ASVs numbers for the green algae (Chlorophyta and Streptophyta). For the Ulvophyceae (Chlorophyta) and Klebsormidiophyceae (Streptophyta), the ITS2 marker resolved the largest numbers of ASVs (14 and 12) among all markers employed (Table 1), whereas the new primer set targeting green algal *rbc*L, GA*rbc*L, excluded the Ulvophyceae. The green algae targeting ITS2 marker, with a total of 234 ASVs, resolved even more ASVs than the 18S marker (Table 1).

**TABLE 1:** Soil algal genera recovered in this study using four different marker genes, their corresponding ASV numbers, and taxonomy.

Phylum	Class	Genera^a^		ASVs^b^			
				23S UPA	*rbcL*	ITS2	18S
Stramenopiles	Xanthophyceae	15	*Asterosiphon*, ***Botrydiopsis***, ***Botrydium***, ***Bumilleria***, ***Bumilleriopsis***, ***Chlorellidium***, ***Heterococcus****, Heterothrix, Mischococcus, Ophiocytium*, ***Pleurochloris***, ***Sphaerosorus***, ***Tribonema***, ***Vaucheria***, ***Xanthonema***	65 (18)	38 (16)	n.a.	21 (9)
	Diatomeae	20	*Amphora, Brachysira, Chamaepinnularia, Craticula, Eolimna, Epithemia, Eucampia, Fistulifera, Gomphoneis, Gomphonema, Halamphora*, ***Hantzschia****, Haslea, Luticola*, ***Mayamaea***, ***Nitzschia****, Pinnularia*, ***Stauroneis****, Surirella*	47 (4)	30 (7)	n.a.	39 (4)
	Eustigmatophyceae	4	*Eustigmatos, Monodopsis, Pseudocharaciopsis, Vischeria (incl. Eustigmatos)*	4 (2)	6 (3)	n.a.	2 (1)
	Total stramenopiles	39		116 (24)	74 (26)	n.a.	62 (14)
Chlorophyta	Chlorophyceae	40	*Actinochloris*, ***Bracteacoccus***, ***Chlamydomonas***, ***Chlorococcum***, ***Chloromonas****, Chlorosarcina*, ***Chlorosarcinopsis***, ***Coelastrella****, Coleochlamys*, ***Deasonia***, ***Desmodesmus***, ***Desmotetra***, ***Dictyococcus***, ***Eubrownia***, ***Fasciculochloris****, Haematococcus*, ***Halochlorella****, Hemiflagellochloris, Heterochlamydomonas, Heterotetracystis, Hormotilopsis, Lobomonas*, ***Macrochloris****, Microglena*, ***Neochloris***, ***Neochlorosarcina***, ***Neocystis****, Neospongiococcum, Planktosphaeria*, ***Pleurastrum***, ***Protosiphon***, ***Pseudomuriella***, ***Rotundella****, Scenedesmus*, ***Spongiochloris****, Spongiococcum*, ***Spongiosarcinopsis***, ***Tetracystis****, Tetradesmus, Vitreochlamys*	31 (10)	51 (23)	132 (27)	109 (42)
	Trebouxiophyceae	28	*Apatococcus, Auxenochlorella*, ***Chlorella***, ***Chloroidium***, ***Coccomyxa***, ***Deuterostichococcus***, ***Diplosphaera***, ***Edaphochlorella****, Edaphochloris, Elliptochloris, Endolithella*, ***Laetitia****, Lobosphaeropsis*, ***Meyerella****, Micractinium*, ***Muriella***, ***Myrmecia***, ***Nannochloris***, ***Parietochloris***, ***Pseudochlorella****, Pseudococcomyxa*, ***Pseudostichococcus****, Raphidonema*, ***Stichococcus***, ***Trebouxia****, Tritostichococcus*, ***Watanabea****, Xerochlorella*	13 (7)	21 (9)	76 (22)	48 (23)
	Ulvophyceae	6	***Desmochloris, Fernandinella, Planophila, Scotinosphaera, Tupiella, Ulothrix***	2 (1)	0	14 (2)	9 (7)
	Pedinophyceae	1	*Pedinomonas*		3 (0)		
Streptophyta	Klebsormidiophyceae	2	***Klebsormidium, Streptosarcina***	5 (4)	2 (1)	12 (4)	2 (2)
	Zygnematophyceae	2	*Cylindrocystis, Mesotaenium*				2 (1)
	Total green algae	79		51 (22)	77 (33)	234 (55)	170 (75)
Bacteria	Cyanobacteria	16	*Allocoleopsis, Anabaena, Calothrix, Crinalium, Cylindrospermum, Drouetiella, Microcoleus, Nodosilinea, Nodularia, Nostoc, Oscillatoria, Phormidium, Pseudanabaena, Romeria, Trichormus, Trichotorquatus*	82 (8)	n.a.	n.a.	n.a.
	Grand total	134		249 (54)	151 (59)	234 (55)	232 (89)

*^a^*Genus names in bold mark those genera that were recovered by two or more markers.

*^b^*Only those ASVs are recorded that had close references in the DDBJ/EMBL/GenBank databases, i.e., *NB* ≥ 1.75. In brackets, number of ASVs with maximal identity with a reference sequence (*NB* ≥ 1.83).

**FIGURE 6:**
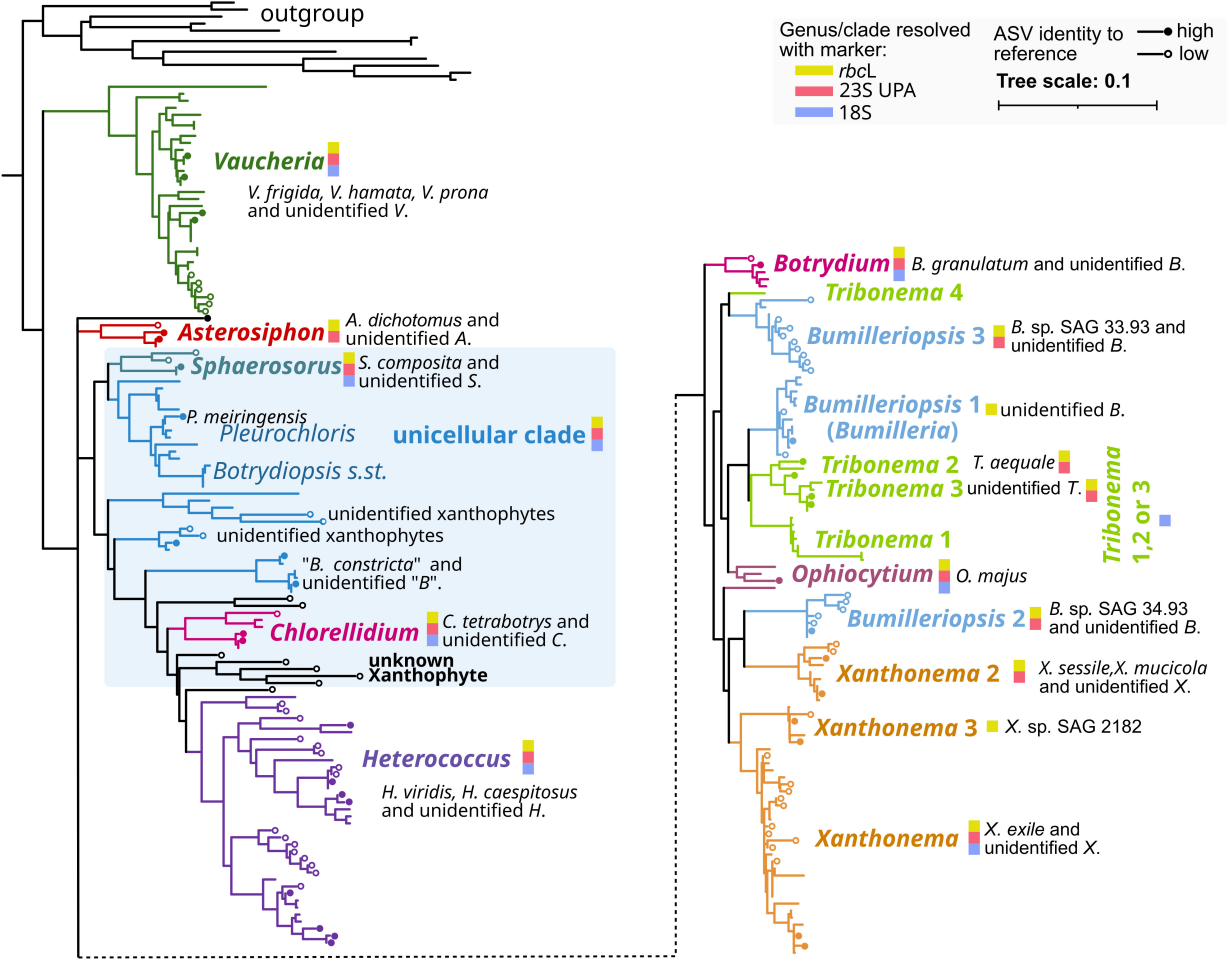
Simplified maximum-likelihood phylogeny based on *rbc*L gene sequences from the Xanthophyceae. The Xanthophyceae ASVs recovered in this study were added via phylogenetic placement. Reference sequences to which ASVs were closely connected, and end points of lines without closer reference are marked by filled and empty circles, respectively. Clades and genera resolved by at least one of the three markers employed are indicated by color. The detailed phylogenies are provided in the Supplementary file S3.

Previous studies, which employed 18S as the only marker for eukaryotes (Aslani et al., 2024; Zhang and Lv, 2022), may have underestimated the Xanthophyceae diversity in the soils of arable land, while that of the green algae dominated (Lukyanov et al., 2024; Zhang and Lv, 2022). However, the availability of appropriate 23S UPA references required for species identification was particularly low for the Xanthophyceae and the Eustigmatophyceae, which made assignment to genera virtually impossible. Consequently, we sequenced a range of 116 Xanthophyceae and nine Eustigmatophyceae strains representing various morphotypes and morphologically identified genera, for their 23S UPA region using the Sanger method (Supplementary Table S1, sheet 4). After adding the new sequences to the GenBank flat file for local queries, the BLAST queries distributed the ASVs across 15 genera of Xanthophyceae and three genera of Eustigmatophyceae (Figure 6 and Supplementary file S3). Using PCR primers for metabarcoding that specifically target Xanthophyceae (Rybalka et al., 2023) may be an appropriate approach to optimize species recovery in this group. Members of the class may also be difficult to recover in cultures. Only a few Xanthophyceae, such as *Bumilleriopsis*, *Botrydiopsis, Heterococcus*, and *Xanthonema*, have previously been identified in culture-based studies of arable land soil algae (Lin et al., 2013; Lukesova, 1993; Zancan et al., 2006). Specifically, the genus *Vaucheria*, which we observed forming mass developments on bare soils in March and November, with its abundant filaments clearly visible in the field (Supplementary Figure S1), must have escaped previous culture-based studies. In contrast to most other Xanthophyceae, *Vaucheria* requires liquid media for maintenance (Hoshino et al., 2024; Schagerl and Kerschbaumer, 2009).

The newly developed primer combination XD*rbc*L, designed to preferentially target a stretch of the *rbc*L gene in the stramenopile algae, and despite amplifying some non-targeted green algae, this marker facilitated the species identification not only in the Xanthophyceae but also the Diatomeae and Eustigmatophyceae (Table 1). Many common soil Diatomeae, such as the genera *Hantzschia*, *Luticula*, and *Mayamaea*, were recovered with all three markers, i.e., the *rbc*L, 23S UPA, and the 18S. However, the genus *Eolimna*, which was shown to be important in arable lands (Heger et al., 2012), was only found using the XD*rbc*L primer combination (Table 1 and Supplementary Table S1). For the recovered Diatomeae *rbc*L ASVs, there were often only low sequence similarities with closest references (low *NB* values), despite *rbc*L sequences have widely been used for diatom identification in freshwater environments. A low representation of terrestrial diatoms in GenBank may account for this, in contrast to environmental studies from freshwater or marine environments (Rimet et al., 2019).

For Cyanobacteria, our metabarcoding approach recovered most of the genera reported in previous culture-based studies (Lukesova, 1993; Zancan et al., 2006). For soil Cyanobacteria in arable land, all previous studies have used 16S rDNA metabarcoding. In those studies, Cyanobacteria constituted only a minor fraction of the bacterial community, typically ranging from 0.01 to 4.5% (Chen et al., 2016; Lauber et al., 2013), and on one occasion reached 9.9% (Lukyanov et al., 2024). However, analyses using the 23S UPA marker indicate that Cyanobacteria account for nearly 50% of the soil algae communities (Lentendu et al., 2014). The low overall abundance of Cyanobacteria reported in previous studies may also be attributable to soil sampling depth and sampling date. The highest cyanobacterial abundance (max. 9.9% relative abundance) was measured by sampling the top five centimeters of soil (Lukyanov et al., 2024). Other studies, sampling the top 13–20 cm of soil, found Cyanobacteria accounting for only a small fraction of the total reads (Chen et al., 2016; Hartmann et al., 2015; Manoharan et al., 2017; Romdhane et al., 2022). Sampling in summer, however, resulted in Cyanobacteria being more abundant (French et al., 2017; Lauber et al., 2013) than in studies that sampled in winter or early spring (Hartmann et al., 2015; Romdhane et al., 2022).

### Phylogenetic diversity of Xanthophyceae ASVs

In our study, the Xanthophyceae, along with the Cyanobacteria, were one of the most important groups of soil algae, with respect to total numbers of ASVs and due to their diversity significantly impacted across environmental changes. Therefore, we asked about the phylogenetic diversity of the recovered genotypes. To assess the phylogenetic diversity of the recovered genotypes, the ASV sequences of the *rbc*L and 18S markers were added to core reference phylogenies using phylogenetic placement. It is an approach that finds the most probable attachment points for short ASV sequences on a pre-existing (“backbone”) phylogeny, based on almost full-length *rbc*L gene sequences, while keeping the tree topology constant (Figure 6 and Supplementary file S3). In the 23S UPA dataset, the reference and ASV sequences were about the same length (368 nucleotides). The 23S UPA reference dataset was primarily based on 126 newly determined sequences from culture strains of Xanthophyceae (Supplementary Table S1, sheet 4). Those strains covered a wide array of genera representing all growth forms of the class (Rybalka et al., 2020). The *rbc*L reference sequence dataset also included several newly determined sequences from culture strains representing unicellular xanthophytes, *Heterococcus*, and five new filamentous isolates from arable fields, which were identified morphologically as *Tribonema* or *Xanthonema* (Supplementary Table S1, sheet 4). It also included the strain CCAP 806/2 *Botrydiopsis arhiza*, representing the taxonomic type of *Botrydiopsis*. Consequently, the *rbc*L backbone phylogeny to which the ASVs were added included all known lineages of the Xanthophyceae (Rybalka et al., 2020).

ASVs with the highest identity to a reference sequence (*NB* = 1.85) had zero internal branch length distance from their closest reference strain, confirming their identification based on BLAST sequence comparisons. ASVs with low sequence identity to a reference sequence were separated from their closest relatives by internal branch lengths, i.e., they were either within a certain clade or genus or were positioned independently of currently known clades and genera (Figure 6 and Supplementary file S3).

There were four groups in the Xanthophyceae phylogenies, which included most of the ASVs across the three markers, 23S UPA, *rbc*L, and 18S. These groups consisted of the clades representing the genera *Heterococcus*, *Vaucheria, Xanthonema* and a paraphyletic array of lineages, referred to here as the “unicellular clade,” centered around the unicellular genera *Botrydiopsis s.str.*, *Chlorellidium*, *Pleurochloris*, and *Sphaerosorus* (Figure 6 and Supplementary file S3). Additionally, the genus *Bumilleriopsis* represented as a paraphyletic array of three independent lineages, was another genus with a substantial number of ASVs assigned, but only with the 23S UPA marker. It means that the majority of Xanthophyceae ASVs our study retrieved from arable lands belonged to these five groups and genera of the Xanthophyceae *rbc*L phylogeny (Figure 6 and Supplementary file S3). The *rbc*L ASVs of *Vaucheria*, thanks to the good availability of reference sequences with the *rbc*L marker, were distributed on the three species, *V. frigida*, *V. hamata*, and *V. prona*. However, one clade contained reference sequences from two species, *V. hamata* and *V. terrestris*, making species-level identification unclear. Furthermore, there was a clade of six ASVs within *Vaucheria*, including the most abundant ASV_1, ASV_3, and ASV_9, which remained unidentified at the species level due to the lack of closer references. In the *rbc*L phylogeny, the strain CCAP 806/2 *B. arhiza* formed together with strains assigned to *B. alpina* a lineage by its own (Supplementary file S3). We suggest considering only this lineage to represent the genus *Botrydiopsis*. Therefore, other lineages with yet unidentified xanthophytes may represent additional genera not yet taxonomically described. In the 18S rDNA phylogeny, *Heterococcus* was inserted into the “unicellular clade,” but there was no bootstrap support for this paraphyletic arrangement. The 18S phylogeny was somewhat exceptional, as most of the ASVs were positioned within clades defined as genera, where they mostly had zero or only short branch lengths to their closest references.

We conclude that our study recovered an expanded diversity of the Xanthophyceae. It entailed not only a large fraction of all currently known genera of the class but also comprised all the growth forms of the Xanthophyceae. Despite using three different markers, the diversity of recovered Xanthophyceae was still not adequately represented by available reference sequences. A large proportion of ASVs were attached to a paraphyletic array of putatively unicellular lineages. They may represent genera and species beyond those currently defined.

## Conclusion and outlook

Our work may serve as a pilot study because it provided initial data on the genotypic diversity of soil algae in Central European arable land and the key factors that determine it. Across multiple markers, our amplicon-based metabarcoding approach revealed dynamics in the genotypic composition of soil algal communities. The key groups in the surface soils of arable land were Chlorophyceae and Trebouxiophyceae within the Chlorophyta, and Xanthophyceae and Diatomeae within the Stramenopiles, together with the Cyanobacteria. These soil algal groups responded sensitively to environmental disturbances associated with the three principal time points determined by the winter wheat’s phenology, contrasting management systems, and variation in the produced crop (wheat versus corn). Other algal groups, the Eustigmatophyceae (Stramenopiles) and Klebsormidiophyceae (Streptophyta), had only a few ( ≤ five) ASVs per sample and remained invariant to those disturbances, except for Klebsormidiophyceae, which were not found in the samples at the March time point. The surface soils of arable land constitute a challenging environment that undergoes constant change and disturbance from agricultural practices. DNA metabarcoding revealed Xanthophyceae and Diatomeae as pioneering algal groups that prefer high bare-soil availability following harvest, tillage, and sowing of a new crop during prolonged periods of low temperatures in early spring. In contrast, the green algae and Cyanobacteria appeared well adapted to higher summer temperatures with low bare-soil availability due to the dense growth of the crop plants.

Our study was limited to a small number of arable fields (*n* = 5) where wheat was the primary crop under the organic management system. At a single (July) sampling point, a small number of geographically neighboring fields with wheat (*n* = 3) or corn (*n* = 2) grown under conventional management were investigated for comparison. Despite these limitations, there was a significant increase in soil algal diversity (ASV counts and Shannon diversity) under the conventional management system compared with the organic management system, whereas the different crop plants had almost no significant effects on soil algal composition. Numerous additional factors related to agricultural practices, which are difficult to disentangle, may have influenced these findings. Our study also revealed several environmental parameters as significant determinants of soil algal community structure, including pH, temperature, and nitrogen content. Some species were even found to thrive under elevated salt content, e.g., the green alga *Desmochloris halophila* (Ulvophyceae). No margin effect for soil algae was found, in contrast to the distinct diversities found for field interior and margin with arable plants (Gayer et al., 2021; Marshall, 2004). Future studies need to test individual factors of agricultural management, such as tillage and fertilization, and compare management systems (i.e., conventional, organic, bio-dynamic) more closely, as well as different crop plant production and soil types, preferably in a *ceteris paribus* manner with sampling from agricultural long-term trials (e.g., Krause et al., 2025; Möller et al., 2024).

We show that for the Xanthophyceae and green algae, two major groups in arable soil systems, multiple markers targeting specific algal groups are required to optimize phylogenetic resolution and increase the number of recovered genotypes (ASVs). For the Xanthophyceae, several genotype lineages were not clearly assignable in the phylogenetic analyses due to a lack of appropriate close references, suggesting that the class’s diversity in arable land surface soils may exceed what is currently known for the class. This demonstrates that future studies on soil algae from arable soils should be complemented by new isolates of soil algal strains to serve as the references needed for improved identification of terrestrial Xanthophyceae. New cultured isolates are also required to enable transcriptomic analyses (e.g., Bierenbroodspot et al., 2024) and to improve understanding of phylogenetic relationships within the Xanthophyceae.

## Data Availability

All sequence data generated from the paired-end approach have been deposited in NCBI Sequence Read Archive database under the accession number PRJNA1258062. All the sequences from Sanger sequencing can be accessed from DDBJ/EMBL/GenBank databases under the accession numbers PV115068-PV115193, PV617327, PV637755-PV637761, which are also given in Supplementary Table S1, Sheet 4.

## Footnotes

1
https://rdocumentation.org/packages/taxonomizr/versions/0.10.6

## Supplementary material

Supplementary Table S1 Information on the samples, culture strains for sequencing, ASV tables including ASV sequences with taxonomy assigned for the 18S, 23 UPA, ITS2, and *rbc*L (primer pairs GA*rbc*L and XD*rbc*L) markers, statistical analyses (averages, *t*-tests) of ASV counts and relative abundances based for the 18S and 23S UPA markers, Pearson correlations of 23S UPA ASV read counts with abiotic parameters, and PERMANOVA test.

Supplementary Figure S1 **(A–C)** Various views of arable field surface soils; **(D,E)** views of studied wheat fields at the July **(D)**, and at the November (**E**; inset, macroscopic view of *Vaucheria* filaments) time points; **(F)** a soil surface sample taken with a teaspoon with a cyanobacterial biofilm on top; **(G,H)** Microscopic views of filamentous Cyanobacteria; **(G)** *Cylindrospermum* sp.; **(E)** *Microcoleus* or *Coleofasciculus* forming bundles; **(I)** location of study area within Germany; **(J)** map of the study area with studied arable fields marked.

Supplementary Figure S2 Monthly averages of air temperatures and precipitation for the year 2024 in the federal state Hesse, Germany, obtained from the *Deutscher Wetterdienst* (weather and climate service for Germany; available at: opendata.dwd.de, accessed 12 February 2026).

Supplementary File S1 Rarefaction curves for all studied samples and five different amplicons, visualized using the R package *mirlyn*. Normalization was performed using the sample with the smallest read count.

Supplementary File S2 Distribution of the algal and cyanobacterial genotypes (ASVs) across the three time points, the contrasting farming management systems, and the two crop plants as based on multiple DNA barcoding markers. The six identified distributional patterns (see text and Figure 5) are indicated at the ASV IDs. Relative abundances (read counts) of the top 50 or 100 ASVs per soil algal group are visualized as heatmaps. Each ASV ID is associated with the accession number of its closest reference sequence, the species identification of the reference, and the normalized bitscore (*NB*) of pairing significance to its closest reference sequence.

Supplementary File S3 Phylogenetic trees of Xanthophyceae, calculated with three different markers using the maximum-likelihood approach. For *rbc*L and 18S, the ASVs were added via phylogenetic placement to the core phylogenies, which were based on nearly full sequences.
