# Supplementary figures and images for: Soil algae in arable land: changes in the genotypic community composition across time points and farming systems—a pilot study
Source: Front Microbiol. 2026 Apr 16;17:1813833. doi: 10.3389/fmicb.2026.1813833 (PMC13128599; doi:10.3389/fmicb.2026.1813833)

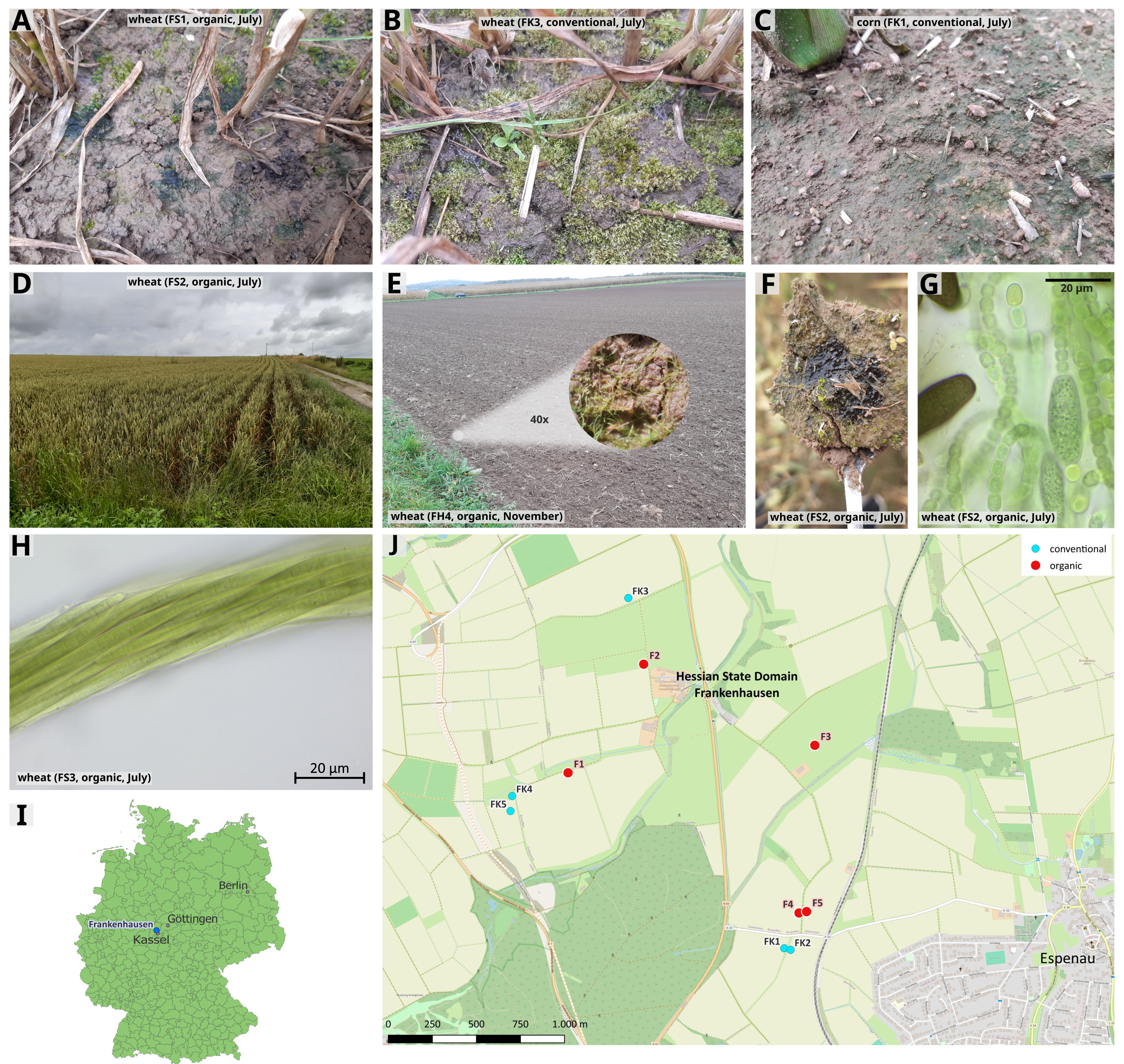

Supplement: Supplementary Figure S1 — (A–C) Various views of arable field surface soils; (D,E) views of studied wheat fields at the July (D), and at the November (E; inset, macroscopic view of Vaucheria filaments) time points; (F) a soil surface sample taken with a teaspoon with a cyanobacterial biofilm on top; (G,H) Microscopic views of filamentous Cyanobacteria; (G) Cylindrospermum sp.; (E) Microcoleus or Coleofasciculus forming bundles; (I) location of study area within Germany; (J) map of the study area with studied arable fields marked. [file Image_1.tiff]

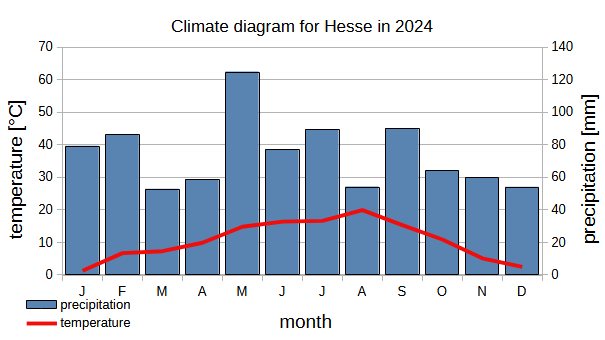

Supplement: Supplementary Figure S2 — Monthly averages of air temperatures and precipitation for the year 2024 in the federal state Hesse, Germany, obtained from the Deutscher Wetterdienst (weather and climate service for Germany; available at: opendata.dwd.de, accessed 12 February 2026). [file Image_2.tif]

## Rarefaction curves for all studied samples

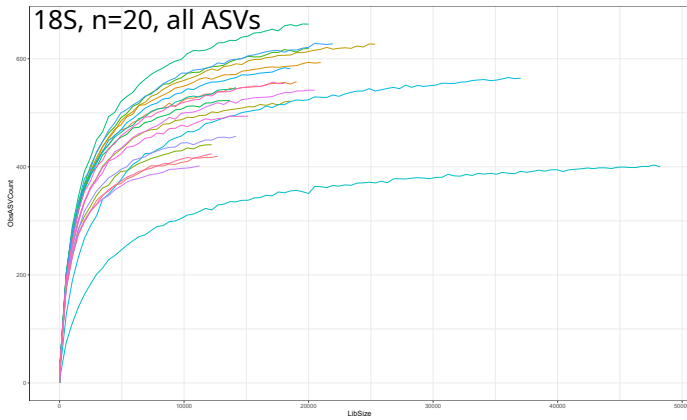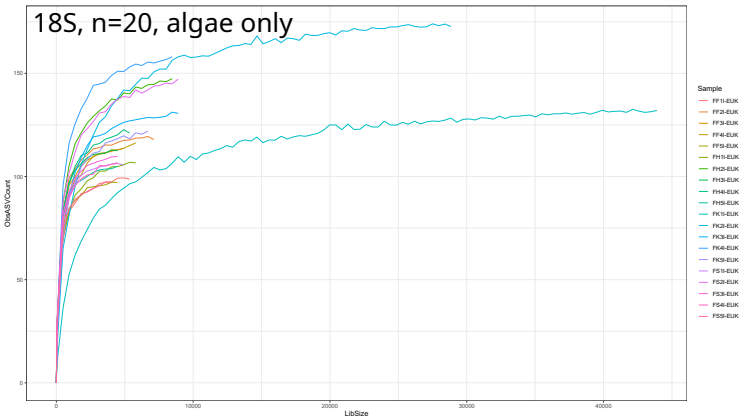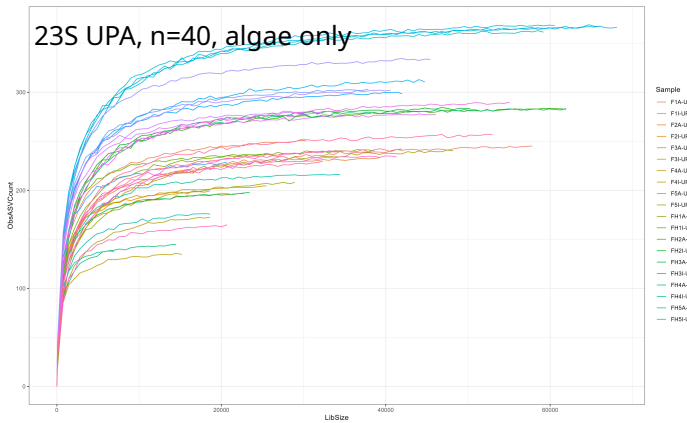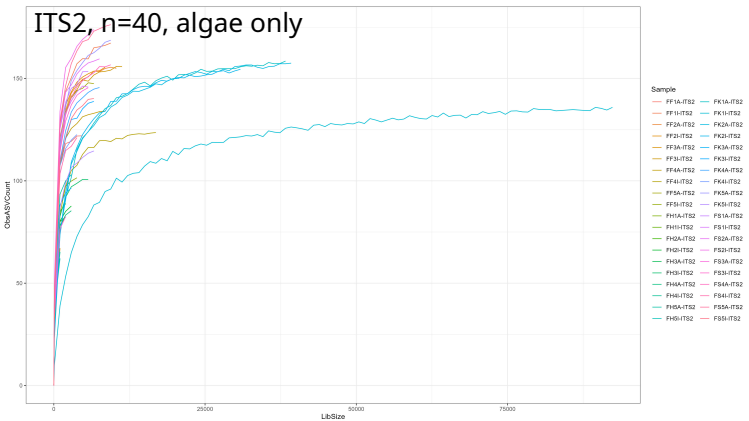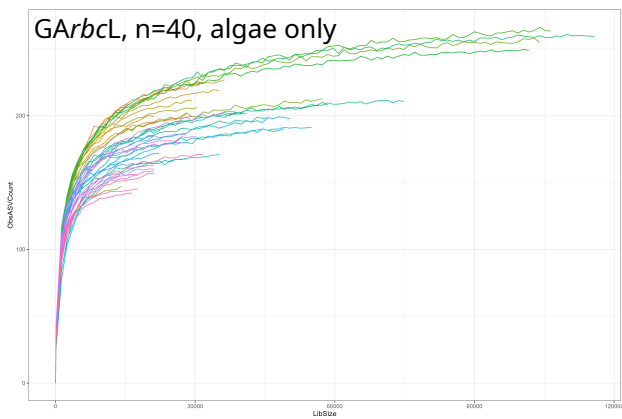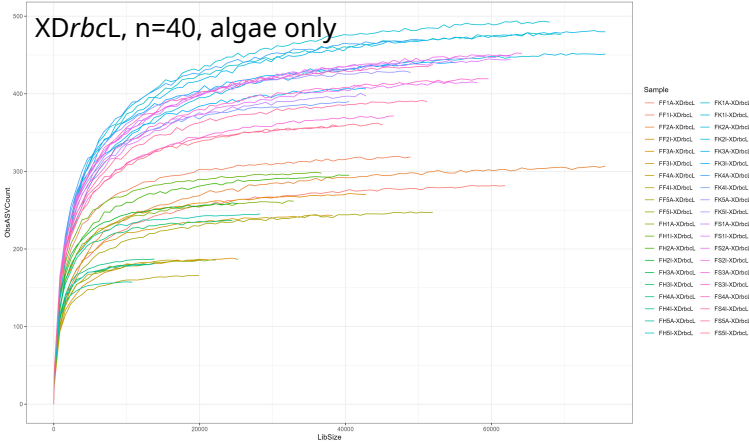

Supplement: Supplementary File S1 — Rarefaction curves for all studied samples and five different amplicons, visualized using the R package mirlyn. Normalization was performed using the sample with the smallest read count. [file Data_Sheet_1.pdf]

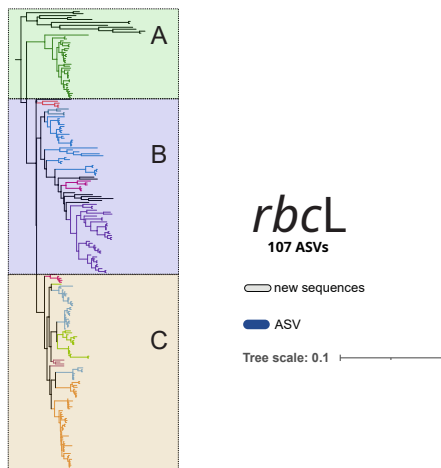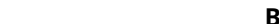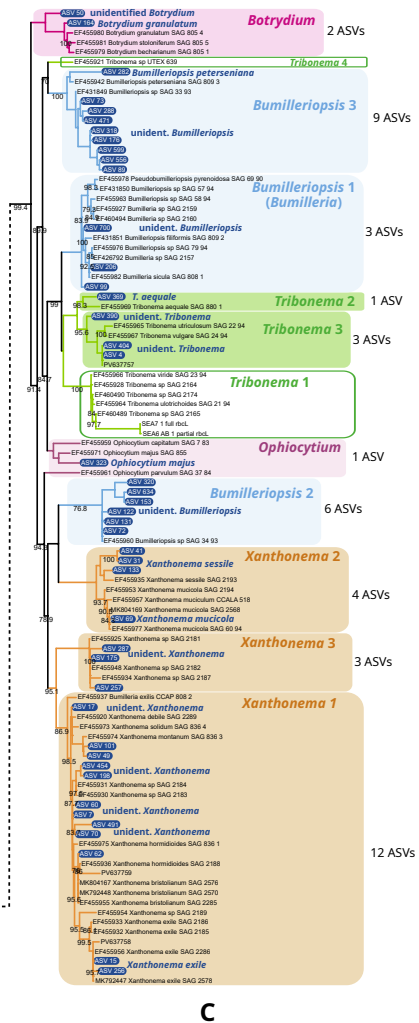

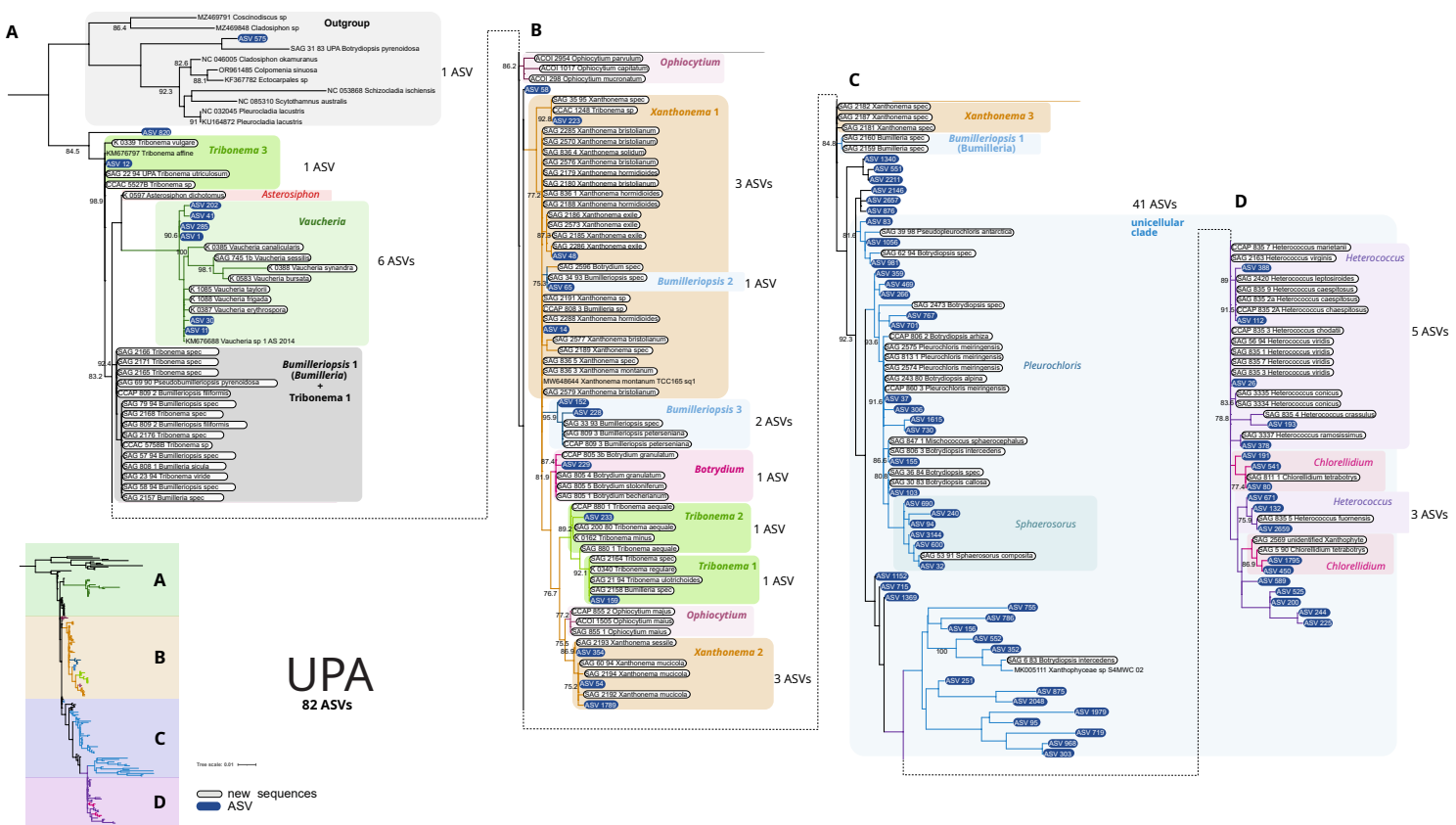

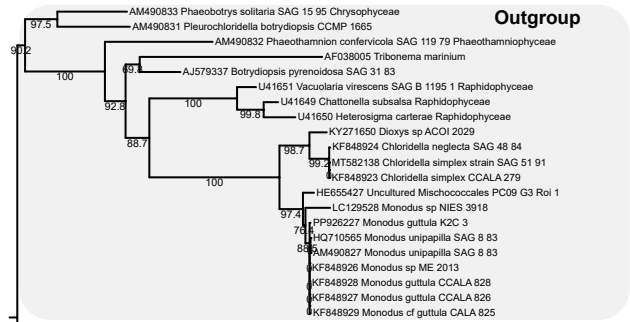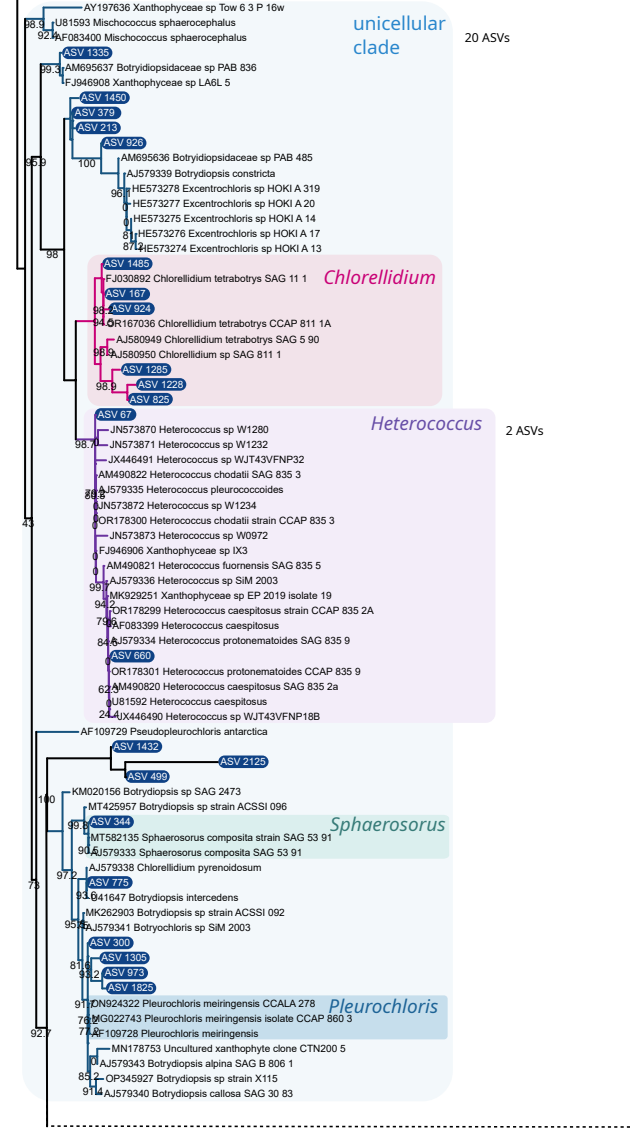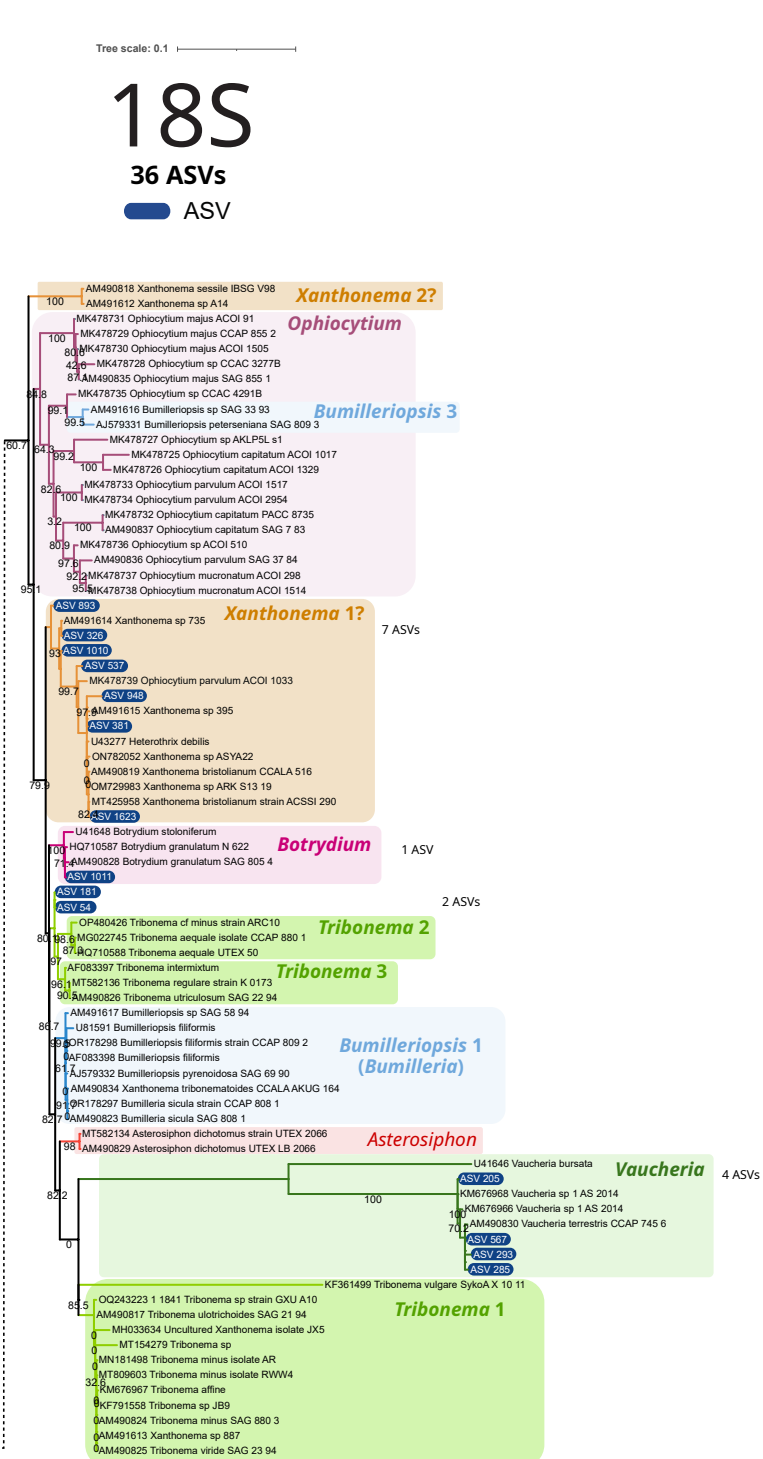

Supplement: Supplementary File S3 — Phylogenetic trees of Xanthophyceae, calculated with three different markers using the maximum-likelihood approach. For rbcL and 18S, the ASVs were added via phylogenetic placement to the core phylogenies, which were based on nearly full sequences. [file Data_Sheet_3.pdf]
