# Supplementary material for: Soil algae in arable land: changes in the genotypic community composition across time points and farming systems—a pilot study
Source: Front Microbiol. 2026 Apr 16;17:1813833. doi: 10.3389/fmicb.2026.1813833 (PMC13128599; doi:10.3389/fmicb.2026.1813833)
Supplement: Supplementary File S2 — Distribution of the algal and cyanobacterial genotypes (ASVs) across the three time points, the contrasting farming management systems, and the two crop plants as based on multiple DNA barcoding markers. The six identified distributional patterns (see text and Figure 5) are indicated at the ASV IDs. Relative abundances (read counts) of the top 50 or 100 ASVs per soil algal group are visualized as heatmaps. Each ASV ID is associated with the accession number of its closest reference sequence, the species identification of the reference, and the normalized bitscore (NB) of pairing significance to its closest reference sequence. [file Data_Sheet_2.pdf]

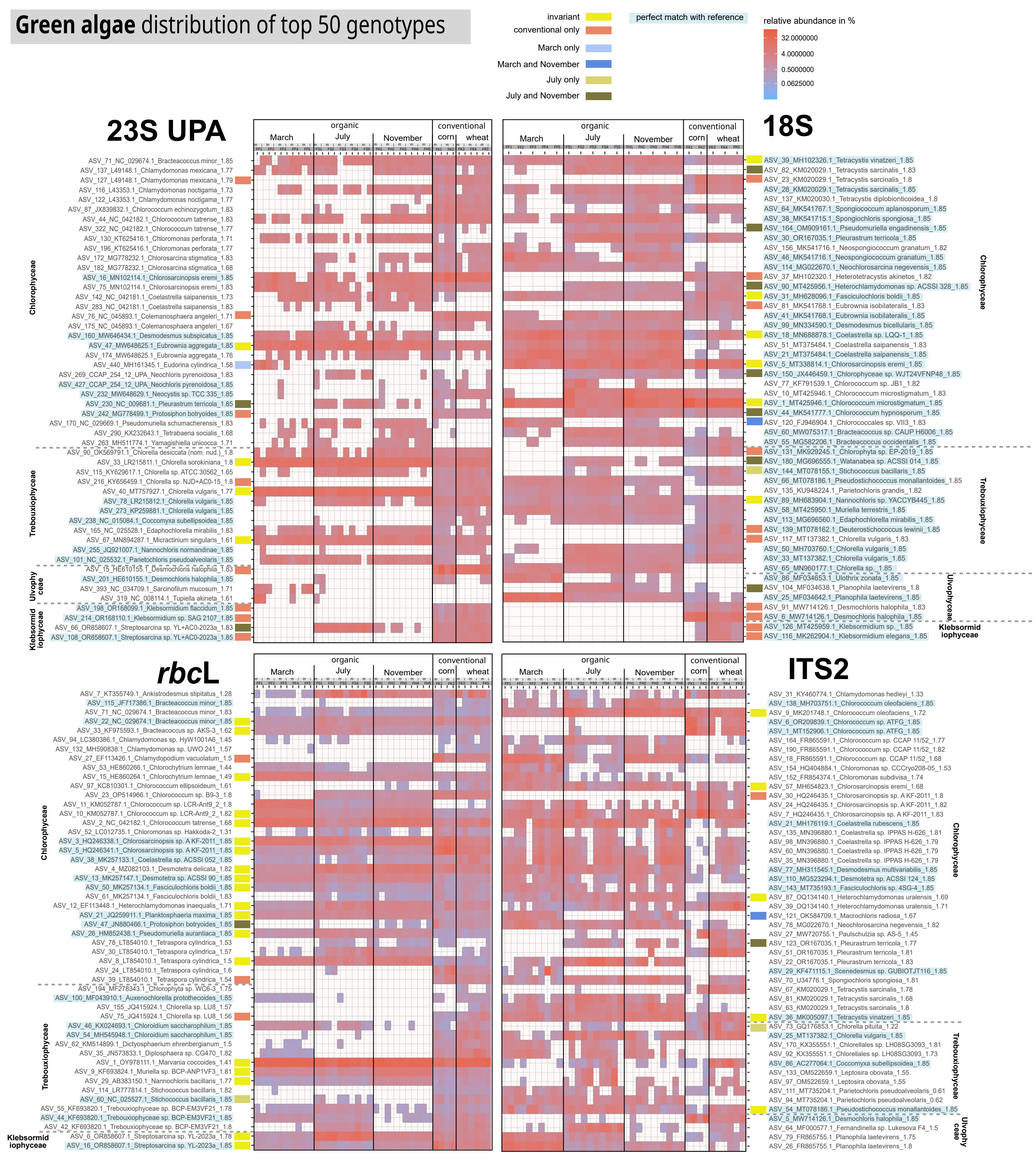

Green algae distribution of top 50 genotypes
invariant
conventional only
March only
March and November
July only
July and November
perfect match with reference
relative abundance in %
32.0000000
4.0000000
0.5000000
0.0625000
23S UPA
18S
rbcL
ITS2
organic
conventional
March
July
November
corn
wheat
FF1 FF2 FF3 FF4 FF5 FS1 FS2 FS3 FS4 FS5 FH1 FH2 FH3 FH4 FH5 FK1 FK2 FK3 FK4 FK5
Chlorophyceae
Trebouxiophyceae
Ulvophyceae
Klebsormidiophyceae
ASV_71_NC_029674.1_Bracteacoccus minor_1.85
ASV_137_L49148.1_Chlamydomonas mexicana_1.77
ASV_127_L49148.1_Chlamydomonas mexicana_1.79
ASV_116_L43353.1_Chlamydomonas noctigama_1.73
ASV_122_L43353.1_Chlamydomonas noctigama_1.77
ASV_87_JX839832.1_Chlorococcum echinozygotum_1.83
ASV_44_NC_042182.1_Chlorococcum tatrense_1.83
ASV_322_NC_042182.1_Chlorococcum tatrense_1.77
ASV_130_KT625416.1_Chloromonas perforata_1.71
ASV_196_KT625416.1_Chloromonas perforata_1.77
ASV_172_MG778232.1_Chlorosarcina stigmatica_1.83
ASV_182_MG778232.1_Chlorosarcina stigmatica_1.68
ASV_16_MN102114.1_Chlorosarcinopsis eremi_1.85
ASV_75_MN102114.1_Chlorosarcinopsis eremi_1.83
ASV_142_NC_042181.1_Coelastrella saipanensis_1.73
ASV_283_NC_042181.1_Coelastrella saipanensis_1.83
ASV_76_NC_045893.1_Colemanosphaera angeleri_1.71
ASV_175_NC_045893.1_Colemanosphaera angeleri_1.67
ASV_160_MW646434.1_Desmodesmus subspicatus_1.85
ASV_47_MW648625.1_Eubrownia aggregata_1.85
ASV_174_MW648625.1_Eubrownia aggregata_1.76
ASV_440_MH161345.1_Eudorina cylindrica_1.58
ASV_269_CCAP_254_12_UPA_Neochloris pyrenoidosa_1.83
ASV_427_CCAP_254_12_UPA_Neochloris pyrenoidosa_1.85
ASV_232_MW648629.1_Neocystis sp. TCC 335_1.85
ASV_230_NC_009681.1_Pleurastrum terricola_1.85
ASV_242_MG778499.1_Protosiphon botryoides_1.85
ASV_170_NC_029669.1_Pseudomuriella schumacherensis_1.83
ASV_290_KX232643.1_Tetrabaena socialis_1.68
ASV_263_MH511774.1_Yamagishiella unicocca_1.71
ASV_90_OK569791.1_Chlorella desiccata (nom. nud.)_1.8
ASV_33_LR215811.1_Chlorella sorokiniana_1.8
ASV_115_KY629617.1_Chlorella sp. ATCC 30562_1.65
ASV_216_KY656459.1_Chlorella sp. NJD+AC0-15_1.8
ASV_40_MT757927.1_Chlorella vulgaris_1.77
ASV_78_LR215812.1_Chlorella vulgaris_1.85
ASV_273_KP259881.1_Chlorella vulgaris_1.85
ASV_238_NC_015084.1_Coccomyxa subellipsoidea_1.85
ASV_165_NC_025528.1_Edaphochlorella mirabilis_1.83
ASV_67_MN894287.1_Micractinium singularis_1.61
ASV_255_JQ921007.1_Nannochloris normandinae_1.85
ASV_101_NC_025532.1_Parietochloris pseudoalveolaris_1.85
ASV_15_HE610155.1_Desmochloris halophila_1.83
ASV_201_HE610155.1_Desmochloris halophila_1.85
ASV_393_NC_034709.1_Sarcinofilum mucosum_1.71
ASV_319_NC_008114.1_Tupiella akineta_1.61
ASV_198_OR168099.1_Klebsormidium flaccidum_1.85
ASV_214_OR168110.1_Klebsormidium sp. SAG 2107_1.85
ASV_66_OR858607.1_Streptosarcina sp. YL+AC0-2023a_1.83
ASV_108_OR858607.1_Streptosarcina sp. YL+AC0-2023a_1.85
ASV_39_MH102326.1_Tetracystis vinatzeri_1.85
ASV_82_KM020029.1_Tetracystis sarcinalis_1.83
ASV_23_KM020029.1_Tetracystis sarcinalis_1.8
ASV_28_KM020029.1_Tetracystis sarcinalis_1.85
ASV_137_KM020030.1_Tetracystis diplobionticoidea_1.8
ASV_64_MK541767.1_Spongiococcum aplanosporum_1.85
ASV_38_MK541715.1_Spongiochloris spongiosa_1.85
ASV_164_OM909161.1_Pseudomuriella engadinensis_1.85
ASV_30_OR167035.1_Pleurastrum terricola_1.85
ASV_156_MK541716.1_Neospongiococcum granatum_1.82
ASV_46_MK541716.1_Neospongiococcum granatum_1.85
ASV_114_MG022670.1_Neochlorosarcina negevensis_1.85
ASV_37_MH102320.1_Heterotetracystis akinetos_1.82
ASV_90_MT425956.1_Heterochlamydomonas sp. ACSSI 328_1.85
ASV_31_MH628096.1_Fasciculochloris boldii_1.85
ASV_81_MK541768.1_Eubrownia isobilateralis_1.83
ASV_41_MK541768.1_Eubrownia isobilateralis_1.85
ASV_99_MN334590.1_Desmodesmus bicellularis_1.85
ASV_18_MN688878.1_Coelastrella sp. LQQ-1_1.85
ASV_51_MT375484.1_Coelastrella saipanensis_1.83
ASV_21_MT375484.1_Coelastrella saipanensis_1.85
ASV_5_MT338814.1_Chlorosarcinopsis eremi_1.85
ASV_150_JX446459.1_Chlorophyceae sp. WJT24VFNP48_1.85
ASV_77_KF791539.1_Chlorococcum sp. JB1_1.82
ASV_10_MT425946.1_Chlorococcum microstigmatum_1.83
ASV_1_MT425946.1_Chlorococcum microstigmatum_1.85
ASV_44_MK541777.1_Chlorococcum hypnosporum_1.85
ASV_120_FJ496904.1_Chlorococcales sp. VII3_1.83
ASV_60_MW075317.1_Bracteacoccus sp. CAUP H6006_1.85
ASV_55_MG582206.1_Bracteacoccus occidentalis_1.85
ASV_131_MK929245.1_Chlorophyta sp. EP-2019_1.85
ASV_180_MG696555.1_Watanabea sp. ACSSI 014_1.85
ASV_144_MT078155.1_Stichococcus bacillaris_1.85
ASV_66_MT078186.1_Pseudostichococcus monallantoides_1.85
ASV_135_KU948224.1_Parietochloris grandis_1.82
ASV_89_MH683904.1_Nannochloris sp. YACCYB445_1.85
ASV_58_MT425950.1_Muriella terrestris_1.85
ASV_113_MG696560.1_Edaphochlorella mirabilis_1.85
ASV_139_MT078162.1_Deuterostichococcus lewinii_1.85
ASV_117_MT137382.1_Chlorella vulgaris_1.83
ASV_50_MH703760.1_Chlorella vulgaris_1.85
ASV_33_MT137382.1_Chlorella vulgaris_1.85
ASV_65_MN960177.1_Chlorella sp._1.85
ASV_86_MF034653.1_Ulothrix zonata_1.85
ASV_104_MF034638.1_Planophila laetevirens_1.8
ASV_25_MF034642.1_Planophila laetevirens_1.85
ASV_91_MW714126.1_Desmochloris halophila_1.83
ASV_6_MW714126.1_Desmochloris halophila_1.85
ASV_126_MT425979.1_Klebsormidium sp._1.85
ASV_116_MK262904.1_Klebsormidium elegans_1.85
ASV_7_KT355749.1_Ankistrodesmus stipitatus_1.28
ASV_115_JF717386.1_Bracteacoccus minor_1.85
ASV_71_NC_029674.1_Bracteacoccus minor_1.83
ASV_22_NC_029674.1_Bracteacoccus minor_1.85
ASV_33_KF975593.1_Bracteacoccus sp. AKS-3_1.62
ASV_94_LC380386.1_Chlamydomonas sp. HyW1001A6_1.45
ASV_132_MH590838.1_Chlamydomonas sp. UWO 241_1.57
ASV_27_EF113426.1_Chlamydopodium vacuolatum_1.5
ASV_53_HE860266.1_Chlorochytrium lemnae_1.44
ASV_15_HE860264.1_Chlorochytrium lemnae_1.49
ASV_97_KC810301.1_Chlorococcum ellipsoideum_1.61
ASV_23_OP514966.1_Chlorococcum sp. B9-3_1.8
ASV_11_KM052787.1_Chlorococcum sp. LCR-Ant9_2_1.8
ASV_10_KM052787.1_Chlorococcum sp. LCR-Ant9_2_1.82
ASV_2_NC_042182.1_Chlorococcum tatrense_1.68
ASV_52_LC012735.1_Chloromonas sp. Hakkoda-2_1.31
ASV_3_HQ246338.1_Chlorosarcinopsis sp. A KF-2011_1.85
ASV_5_HQ246341.1_Chlorosarcinopsis sp. A KF-2011_1.85
ASV_38_MK257133.1_Coelastrella sp. ACSSI 052_1.85
ASV_4_MZ082103.1_Desmotetra delicata_1.82
ASV_13_MK257147.1_Desmotetra sp. ACSSI 90_1.85
ASV_50_MK257134.1_Fasciculochloris boldii_1.85
ASV_61_MK257134.1_Fasciculochloris boldii_1.83
ASV_12_EF113448.1_Heterochlamydomonas inaequalis_1.71
ASV_21_JQ259911.1_Planktosphaeria maxima_1.85
ASV_47_JN880466.1_Protosiphon botryoides_1.85
ASV_26_HM852438.1_Pseudomuriella aurantiaca_1.85
ASV_78_LT854010.1_Tetraspora cylindrica_1.53
ASV_30_LT854010.1_Tetraspora cylindrica_1.57
ASV_8_LT854010.1_Tetraspora cylindrica_1.5
ASV_24_LT854010.1_Tetraspora cylindrica_1.6
ASV_39_LT854010.1_Tetraspora cylindrica_1.54
ASV_194_MF278343.1_Chlorophyta sp. WC6-3_1.75
ASV_100_MF043910.1_Auxenochlorella protothecoides_1.85
ASV_155_JQ415924.1_Chlorella sp. LU8_1.57
ASV_75_JQ415924.1_Chlorella sp. LU8_1.56
ASV_46_KX024693.1_Chloroidium saccharophilum_1.85
ASV_54_MH545948.1_Chloroidium saccharophilum_1.85
ASV_62_KM514899.1_Dictyosphaerium ehrenbergianum_1.5
ASV_35_JN573833.1_Diplosphaera sp. CG470_1.82
ASV_1_OY978111.1_Marvania coccoides_1.41
ASV_9_KF693824.1_Muriella sp. BCP-ANP1VF3_1.81
ASV_29_AB383150.1_Nannochloris bacillaris_1.77
ASV_114_LR777814.1_Stichococcus bacillaris_1.82
ASV_60_NC_025527.1_Stichococcus bacillaris_1.85
ASV_55_KF693820.1_Trebouxiophyceae sp. BCP-EM3VF21_1.78
ASV_44_KF693820.1_Trebouxiophyceae sp. BCP-EM3VF21_1.85
ASV_42_KF693820.1_Trebouxiophyceae sp. BCP-EM3VF21_1.8
ASV_6_OR858607.1_Streptosarcina sp. YL-2023a_1.78
ASV_16_OR858607.1_Streptosarcina sp. YL-2023a_1.85
ASV_31_KY460774.1_Chlamydomonas hedleyi_1.33
ASV_138_MH703751.1_Chlorococcum oleofaciens_1.85
ASV_9_MK201748.1_Chlorococcum oleofaciens_1.72
ASV_6_OR209839.1_Chlorococcum sp. ATFG_1.85
ASV_1_MT152906.1_Chlorococcum sp. ATFG_1.85
ASV_164_FR865591.1_Chlorococcum sp. CCAP 11/52_1.77
ASV_190_FR865591.1_Chlorococcum sp. CCAP 11/52_1.82
ASV_18_FR865591.1_Chlorococcum sp. CCAP 11/52_1.68
ASV_154_HQ404884.1_Chloromonas sp. CCCryo208-05_1.53
ASV_152_FR854374.1_Chloromonas subdivisa_1.74
ASV_57_MH654823.1_Chlorosarcinopsis eremi_1.68
ASV_30_HQ246435.1_Chlorosarcinopsis sp. A KF-2011_1.8
ASV_24_HQ246435.1_Chlorosarcinopsis sp. A KF-2011_1.82
ASV_7_HQ246435.1_Chlorosarcinopsis sp. A KF-2011_1.83
ASV_21_MH176119.1_Coelastrella rubescens_1.85
ASV_135_MN396880.1_Coelastrella sp. IPPAS H-626_1.81
ASV_98_MN396880.1_Coelastrella sp. IPPAS H-626_1.79
ASV_60_MN396880.1_Coelastrella sp. IPPAS H-626_1.79
ASV_35_MN396880.1_Coelastrella sp. IPPAS H-626_1.79
ASV_77_MH311545.1_Desmodesmus multivariabilis_1.85
ASV_110_MG523294.1_Desmotetra sp. ACSSI 124_1.85
ASV_143_MT735193.1_Fasciculochloris sp. 4SG-4_1.85
ASV_87_OQ134140.1_Heterochlamydomonas uralensis_1.69
ASV_39_OQ134140.1_Heterochlamydomonas uralensis_1.71
ASV_121_OK584709.1_Macrochloris radiosa_1.67
ASV_78_MG022670.1_Neochlorosarcina negevensis_1.82
ASV_27_MW720755.1_Paulschulzia sp. AS-5_1.45
ASV_123_OR167035.1_Pleurastrum terricola_1.77
ASV_51_OR167035.1_Pleurastrum terricola_1.81
ASV_22_OR167035.1_Pleurastrum terricola_1.83
ASV_29_KF471115.1_Scenedesmus sp. GUBIOTJT116_1.85
ASV_70_U34776.1_Spongiochloris spongiosa_1.81
ASV_67_KM020029.1_Tetracystis sarcinalis_1.78
ASV_81_KM020029.1_Tetracystis sarcinalis_1.68
ASV_63_KM020029.1_Tetracystis sarcinalis_1.8
ASV_36_MK005097.1_Tetracystis vinatzeri_1.85
ASV_73_GQ176853.1_Chlorella pituita_1.22
ASV_25_MT137382.1_Chlorella vulgaris_1.85
ASV_170_KX355551.1_Chlorellales sp. LH08SG3093_1.81
ASV_92_KX355551.1_Chlorellales sp. LH08SG3093_1.73
ASV_86_AC277064.1_Coccomyxa subellipsoidea_1.85
ASV_133_OM522659.1_Leptosira obovata_1.55
ASV_97_OM522659.1_Leptosira obovata_1.55
ASV_111_MT735204.1_Parietochloris pseudoalveolaris_0.61
ASV_94_MT735204.1_Parietochloris pseudoalveolaris_0.62
ASV_54_MT078186.1_Pseudostichococcus monallantoides_1.85
ASV_5_MW714126.1_Desmochloris halophila_1.85
ASV_64_MF000577.1_Fernandinella sp. Lukesova F4_1.5
ASV_79_FR865755.1_Planophila laetevirens_1.75
ASV_26_FR865755.1_Planophila laetevirens_1.8

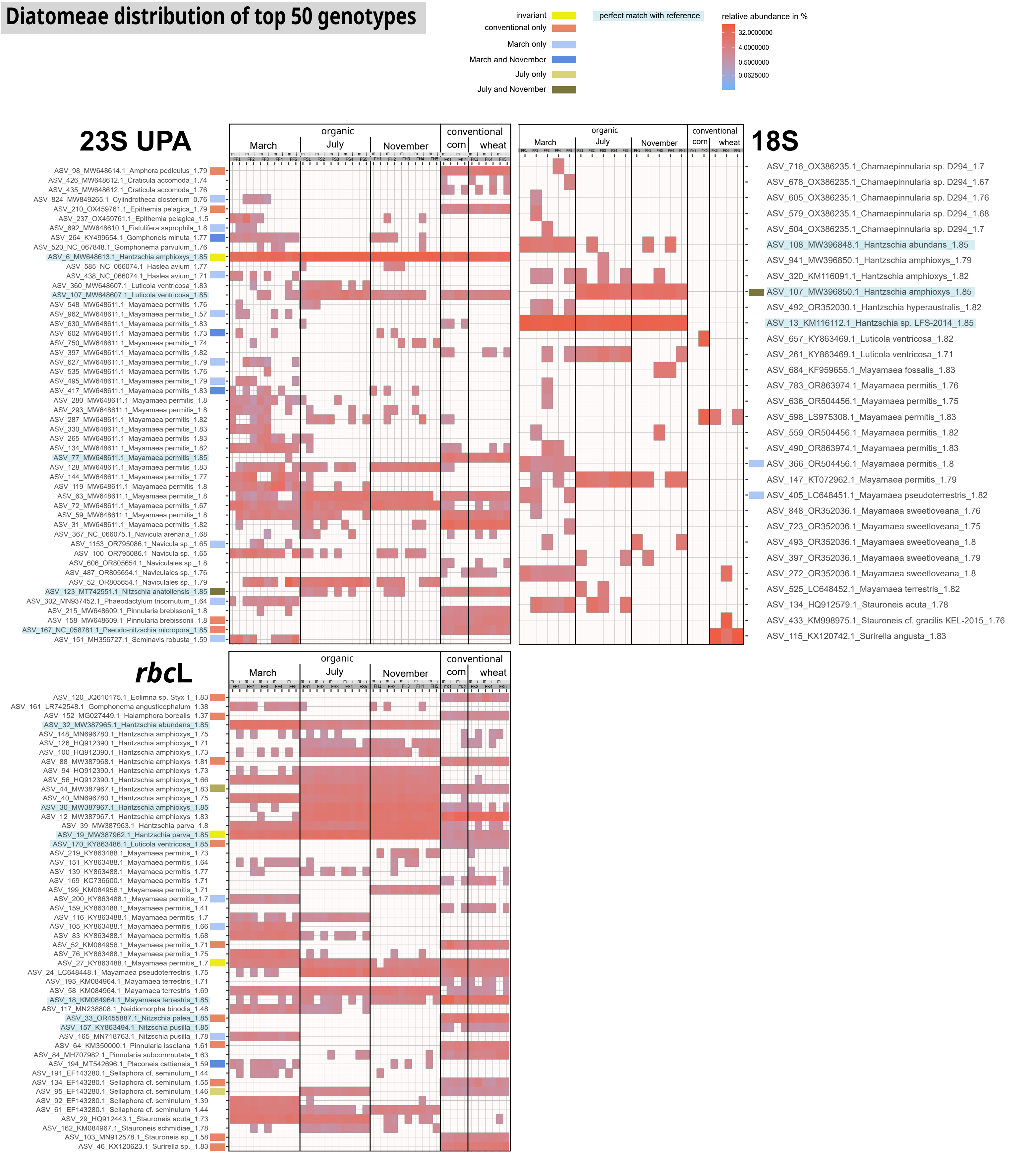

Diatomeae distribution of top 50 genotypes
invariant
conventional only
March only
March and November
July only
July and November
perfect match with reference
relative abundance in %
32.0000000
4.0000000
0.5000000
0.0625000
23S UPA
organic
March
July
November
conventional
corn
wheat
FF1 FF2 FF3 FF4 FF5 FS1 FS2 FS3 FS4 FS5 FH1 FH2 FH3 FH4 FH5 FK1 FK2 FK3 FK4 FK5
ASV_98_MW648614.1_Amphora pediculus_1.79
ASV_426_MW648612.1_Craticula accomoda_1.74
ASV_435_MW648612.1_Craticula accomoda_1.76
ASV_824_MW849265.1_Cylindrotheca closterium_0.76
ASV_210_OX459761.1_Epithemia pelagica_1.79
ASV_237_OX459761.1_Epithemia pelagica_1.5
ASV_692_MW648610.1_Fistulifera saprophila_1.8
ASV_264_KY499654.1_Gomphoneis minuta_1.77
ASV_520_NC_067848.1_Gomphonema parvulum_1.76
ASV_6_MW648613.1_Hantzschia amphioxys_1.85
ASV_585_NC_066074.1_Haslea avium_1.77
ASV_438_NC_066074.1_Haslea avium_1.71
ASV_360_MW648607.1_Luticola ventricosa_1.83
ASV_107_MW648607.1_Luticola ventricosa_1.85
ASV_548_MW648611.1_Mayamaea permitis_1.76
ASV_962_MW648611.1_Mayamaea permitis_1.57
ASV_630_MW648611.1_Mayamaea permitis_1.83
ASV_602_MW648611.1_Mayamaea permitis_1.73
ASV_750_MW648611.1_Mayamaea permitis_1.74
ASV_397_MW648611.1_Mayamaea permitis_1.82
ASV_627_MW648611.1_Mayamaea permitis_1.79
ASV_535_MW648611.1_Mayamaea permitis_1.76
ASV_495_MW648611.1_Mayamaea permitis_1.79
ASV_417_MW648611.1_Mayamaea permitis_1.83
ASV_280_MW648611.1_Mayamaea permitis_1.8
ASV_293_MW648611.1_Mayamaea permitis_1.8
ASV_287_MW648611.1_Mayamaea permitis_1.82
ASV_330_MW648611.1_Mayamaea permitis_1.83
ASV_265_MW648611.1_Mayamaea permitis_1.83
ASV_134_MW648611.1_Mayamaea permitis_1.82
ASV_77_MW648611.1_Mayamaea permitis_1.85
ASV_128_MW648611.1_Mayamaea permitis_1.83
ASV_144_MW648611.1_Mayamaea permitis_1.77
ASV_119_MW648611.1_Mayamaea permitis_1.8
ASV_63_MW648611.1_Mayamaea permitis_1.8
ASV_72_MW648611.1_Mayamaea permitis_1.67
ASV_59_MW648611.1_Mayamaea permitis_1.8
ASV_31_MW648611.1_Mayamaea permitis_1.82
ASV_367_NC_066075.1_Navicula arenaria_1.68
ASV_1153_OR795086.1_Navicula sp._1.65
ASV_100_OR795086.1_Navicula sp._1.65
ASV_606_OR805654.1_Naviculales sp._1.8
ASV_487_OR805654.1_Naviculales sp._1.76
ASV_52_OR805654.1_Naviculales sp._1.79
ASV_123_MT742551.1_Nitzschia anatoliensis_1.85
ASV_302_MN937452.1_Phaeodactylum tricornutum_1.64
ASV_215_MW648609.1_Pinnularia brebissonii_1.8
ASV_158_MW648609.1_Pinnularia brebissonii_1.8
ASV_167_NC_058781.1_Pseudo-nitzschia micropora_1.85
ASV_151_MH356727.1_Seminavis robusta_1.59
18S
organic
March
July
November
conventional
corn
wheat
ASV_716_OX386235.1_Chamaepinnularia sp. D294_1.7
ASV_678_OX386235.1_Chamaepinnularia sp. D294_1.67
ASV_605_OX386235.1_Chamaepinnularia sp. D294_1.76
ASV_579_OX386235.1_Chamaepinnularia sp. D294_1.68
ASV_504_OX386235.1_Chamaepinnularia sp. D294_1.7
ASV_108_MW396848.1_Hantzschia abundans_1.85
ASV_941_MW396850.1_Hantzschia amphioxys_1.79
ASV_320_KM116091.1_Hantzschia amphioxys_1.82
ASV_107_MW396850.1_Hantzschia amphioxys_1.85
ASV_492_OR352030.1_Hantzschia hyperaustralis_1.82
ASV_13_KM116112.1_Hantzschia sp. LFS-2014_1.85
ASV_657_KY863469.1_Luticola ventricosa_1.82
ASV_261_KY863469.1_Luticola ventricosa_1.71
ASV_684_KF959655.1_Mayamaea fossalis_1.83
ASV_783_OR863974.1_Mayamaea permitis_1.76
ASV_636_OR504456.1_Mayamaea permitis_1.75
ASV_598_LS975308.1_Mayamaea permitis_1.83
ASV_559_OR504456.1_Mayamaea permitis_1.82
ASV_490_OR863974.1_Mayamaea permitis_1.83
ASV_366_OR504456.1_Mayamaea permitis_1.8
ASV_147_KT072962.1_Mayamaea permitis_1.79
ASV_405_LC648451.1_Mayamaea pseudoterrestris_1.82
ASV_848_OR352036.1_Mayamaea sweetloveana_1.76
ASV_723_OR352036.1_Mayamaea sweetloveana_1.75
ASV_493_OR352036.1_Mayamaea sweetloveana_1.8
ASV_397_OR352036.1_Mayamaea sweetloveana_1.79
ASV_272_OR352036.1_Mayamaea sweetloveana_1.8
ASV_525_LC648452.1_Mayamaea terrestris_1.82
ASV_134_HQ912579.1_Stauroneis acuta_1.78
ASV_433_KM998975.1_Stauroneis cf. gracilis KEL-2015_1.76
ASV_115_KX120742.1_Surirella angusta_1.83
rbcL
organic
March
July
November
conventional
corn
wheat
ASV_120_JQ610175.1_Eolimna sp. Styx 1_1.83
ASV_161_LR742548.1_Gomphonema angusticephalum_1.38
ASV_152_MG027449.1_Halamphora borealis_1.37
ASV_32_MW387965.1_Hantzschia abundans_1.85
ASV_148_MN696780.1_Hantzschia amphioxys_1.75
ASV_126_HQ912390.1_Hantzschia amphioxys_1.71
ASV_100_HQ912390.1_Hantzschia amphioxys_1.73
ASV_88_MW387968.1_Hantzschia amphioxys_1.81
ASV_94_HQ912390.1_Hantzschia amphioxys_1.73
ASV_56_HQ912390.1_Hantzschia amphioxys_1.66
ASV_44_MW387967.1_Hantzschia amphioxys_1.83
ASV_40_MN696780.1_Hantzschia amphioxys_1.75
ASV_30_MW387967.1_Hantzschia amphioxys_1.85
ASV_12_MW387967.1_Hantzschia amphioxys_1.83
ASV_39_MW387963.1_Hantzschia parva_1.8
ASV_19_MW387962.1_Hantzschia parva_1.85
ASV_170_KY863486.1_Luticola ventricosa_1.85
ASV_219_KY863488.1_Mayamaea permitis_1.73
ASV_151_KY863488.1_Mayamaea permitis_1.64
ASV_139_KY863488.1_Mayamaea permitis_1.77
ASV_169_KC736600.1_Mayamaea permitis_1.71
ASV_199_KM084956.1_Mayamaea permitis_1.71
ASV_200_KY863488.1_Mayamaea permitis_1.7
ASV_159_KY863488.1_Mayamaea permitis_1.41
ASV_116_KY863488.1_Mayamaea permitis_1.7
ASV_105_KY863488.1_Mayamaea permitis_1.66
ASV_83_KY863488.1_Mayamaea permitis_1.68
ASV_52_KM084956.1_Mayamaea permitis_1.71
ASV_76_KY863488.1_Mayamaea permitis_1.75
ASV_27_KY863488.1_Mayamaea permitis_1.7
ASV_24_LC648448.1_Mayamaea pseudoterrestris_1.75
ASV_195_KM084964.1_Mayamaea terrestris_1.71
ASV_58_KM084964.1_Mayamaea terrestris_1.69
ASV_18_KM084964.1_Mayamaea terrestris_1.85
ASV_117_MN238808.1_Neidiomorpha binodis_1.48
ASV_33_OR455887.1_Nitzschia palea_1.85
ASV_157_KY863494.1_Nitzschia pusilla_1.85
ASV_165_MN718763.1_Nitzschia pusilla_1.78
ASV_64_KM350000.1_Pinnularia isselana_1.61
ASV_84_MH707982.1_Pinnularia subcommutata_1.63
ASV_194_MT542696.1_Placoneis cattiensis_1.59
ASV_191_EF143280.1_Sellaphora cf. seminulum_1.44
ASV_134_EF143280.1_Sellaphora cf. seminulum_1.55
ASV_95_EF143280.1_Sellaphora cf. seminulum_1.46
ASV_92_EF143280.1_Sellaphora cf. seminulum_1.39
ASV_61_EF143280.1_Sellaphora cf. seminulum_1.44
ASV_29_HQ912443.1_Stauroneis acuta_1.73
ASV_162_KM084967.1_Stauroneis schmidiae_1.78
ASV_103_MN912578.1_Stauroneis sp._1.58
ASV_46_KX120623.1_Surirella sp._1.83

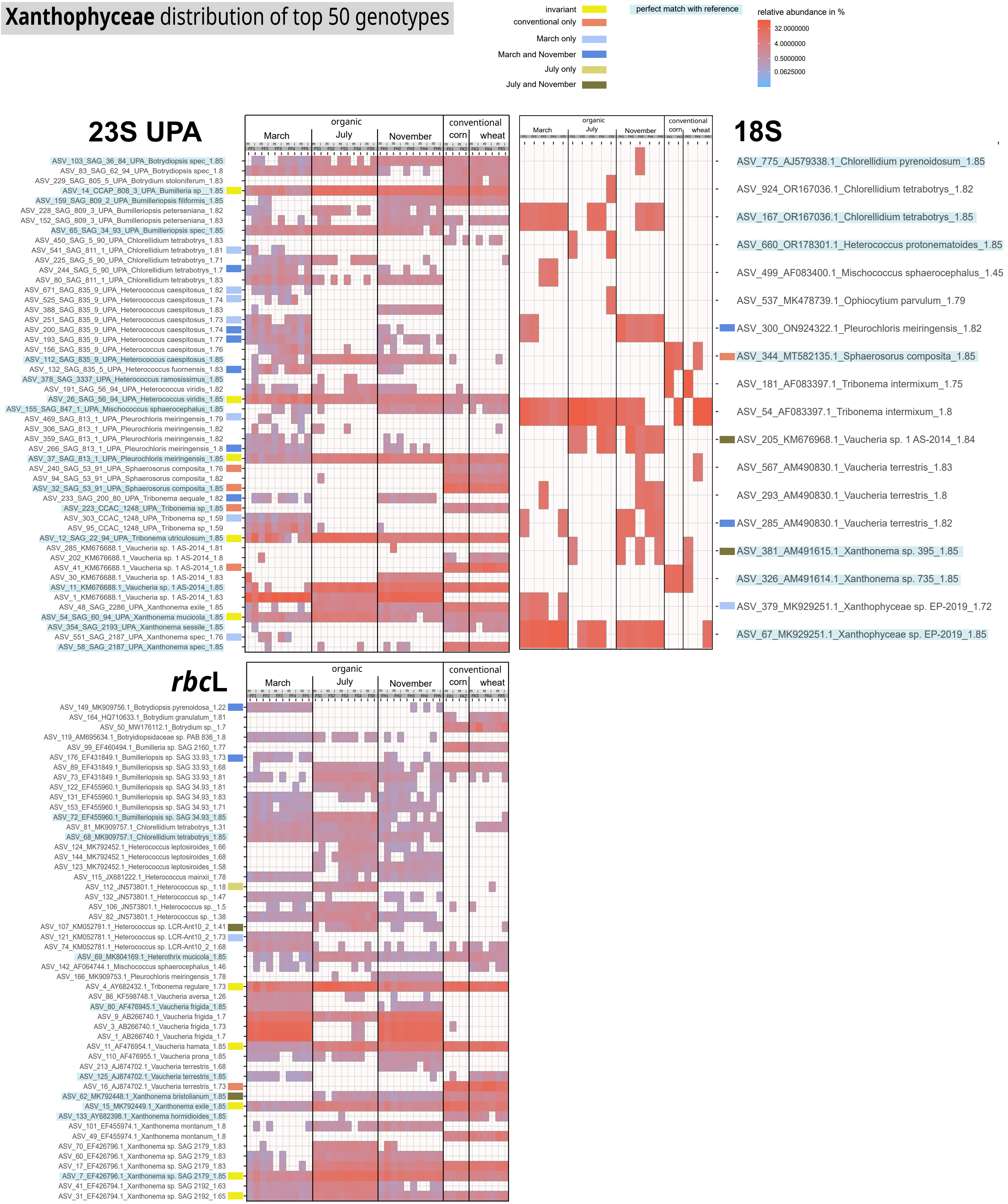

Xanthophyceae distribution of top 50 genotypes
invariant
conventional only
March only
March and November
July only
July and November
perfect match with reference
relative abundance in %
32.0000000
4.0000000
0.5000000
0.0625000
23S UPA
organic
March
July
November
conventional
corn
wheat
FF1 FF2 FF3 FF4 FF5 FS1 FS2 FS3 FS4 FS5 FH1 FH2 FH3 FH4 FH5 FK1 FK2 FK3 FK4 FK5
ASV_103_SAG_36_84_UPA_Botrydiopsis spec_1.85
ASV_83_SAG_62_94_UPA_Botrydiopsis spec_1.8
ASV_229_SAG_805_5_UPA_Botrydium stoloniferum_1.83
ASV_14_CCAP_808_3_UPA_Bumilleria sp._1.85
ASV_159_SAG_809_2_UPA_Bumilleriopsis filiformis_1.85
ASV_228_SAG_809_3_UPA_Bumilleriopsis peterseniana_1.82
ASV_152_SAG_809_3_UPA_Bumilleriopsis peterseniana_1.83
ASV_65_SAG_34_93_UPA_Bumilleriopsis spec_1.85
ASV_450_SAG_5_90_UPA_Chlorellidium tetrabotrys_1.83
ASV_541_SAG_811_1_UPA_Chlorellidium tetrabotrys_1.81
ASV_225_SAG_5_90_UPA_Chlorellidium tetrabotrys_1.71
ASV_244_SAG_5_90_UPA_Chlorellidium tetrabotrys_1.7
ASV_80_SAG_811_1_UPA_Chlorellidium tetrabotrys_1.83
ASV_671_SAG_835_9_UPA_Heterococcus caespitosus_1.82
ASV_525_SAG_835_9_UPA_Heterococcus caespitosus_1.74
ASV_388_SAG_835_9_UPA_Heterococcus caespitosus_1.83
ASV_251_SAG_835_9_UPA_Heterococcus caespitosus_1.73
ASV_200_SAG_835_9_UPA_Heterococcus caespitosus_1.74
ASV_193_SAG_835_9_UPA_Heterococcus caespitosus_1.77
ASV_156_SAG_835_9_UPA_Heterococcus caespitosus_1.76
ASV_112_SAG_835_9_UPA_Heterococcus caespitosus_1.85
ASV_132_SAG_835_5_UPA_Heterococcus fuornensis_1.83
ASV_378_SAG_3337_UPA_Heterococcus ramosissimus_1.85
ASV_191_SAG_56_94_UPA_Heterococcus viridis_1.82
ASV_26_SAG_56_94_UPA_Heterococcus viridis_1.85
ASV_155_SAG_847_1_UPA_Mischococcus sphaerocephalus_1.85
ASV_469_SAG_813_1_UPA_Pleurochloris meiringensis_1.79
ASV_306_SAG_813_1_UPA_Pleurochloris meiringensis_1.82
ASV_359_SAG_813_1_UPA_Pleurochloris meiringensis_1.82
ASV_266_SAG_813_1_UPA_Pleurochloris meiringensis_1.8
ASV_37_SAG_813_1_UPA_Pleurochloris meiringensis_1.85
ASV_240_SAG_53_91_UPA_Sphaerosorus composita_1.76
ASV_94_SAG_53_91_UPA_Sphaerosorus composita_1.82
ASV_32_SAG_53_91_UPA_Sphaerosorus composita_1.85
ASV_233_SAG_200_80_UPA_Tribonema aequale_1.82
ASV_223_CCAC_1248_UPA_Tribonema sp_1.85
ASV_303_CCAC_1248_UPA_Tribonema sp_1.59
ASV_95_CCAC_1248_UPA_Tribonema sp_1.59
ASV_12_SAG_22_94_UPA_Tribonema utriculosum_1.85
ASV_285_KM676688.1_Vaucheria sp. 1 AS-2014_1.81
ASV_202_KM676688.1_Vaucheria sp. 1 AS-2014_1.8
ASV_41_KM676688.1_Vaucheria sp. 1 AS-2014_1.8
ASV_30_KM676688.1_Vaucheria sp. 1 AS-2014_1.83
ASV_11_KM676688.1_Vaucheria sp. 1 AS-2014_1.85
ASV_1_KM676688.1_Vaucheria sp. 1 AS-2014_1.83
ASV_48_SAG_2286_UPA_Xanthonema exile_1.85
ASV_54_SAG_60_94_UPA_Xanthonema mucicola_1.85
ASV_354_SAG_2193_UPA_Xanthonema sessile_1.85
ASV_551_SAG_2187_UPA_Xanthonema spec_1.76
ASV_58_SAG_2187_UPA_Xanthonema spec_1.85
18S
organic
March
July
November
conventional
corn
wheat
ASV_775_AJ579338.1_Chlorellidium pyrenoidosum_1.85
ASV_924_OR167036.1_Chlorellidium tetrabotrys_1.82
ASV_167_OR167036.1_Chlorellidium tetrabotrys_1.85
ASV_660_OR178301.1_Heterococcus protonematoides_1.85
ASV_499_AF083400.1_Mischococcus sphaerocephalus_1.45
ASV_537_MK478739.1_Ophiocytium parvulum_1.79
ASV_300_ON924322.1_Pleurochloris meiringensis_1.82
ASV_344_MT582135.1_Sphaerosorus composita_1.85
ASV_181_AF083397.1_Tribonema intermixum_1.75
ASV_54_AF083397.1_Tribonema intermixum_1.8
ASV_205_KM676968.1_Vaucheria sp. 1 AS-2014_1.84
ASV_567_AM490830.1_Vaucheria terrestris_1.83
ASV_293_AM490830.1_Vaucheria terrestris_1.8
ASV_285_AM490830.1_Vaucheria terrestris_1.82
ASV_381_AM491615.1_Xanthonema sp. 395_1.85
ASV_326_AM491614.1_Xanthonema sp. 735_1.85
ASV_379_MK929251.1_Xanthophyceae sp. EP-2019_1.72
ASV_67_MK929251.1_Xanthophyceae sp. EP-2019_1.85
rbcL
organic
March
July
November
conventional
corn
wheat
ASV_149_MK909756.1_Botrydiopsis pyrenoidosa_1.22
ASV_164_HQ710633.1_Botrydium granulatum_1.81
ASV_50_MW176112.1_Botrydium sp._1.7
ASV_119_AM695634.1_Botryidiopsidaceae sp. PAB 836_1.8
ASV_99_EF460494.1_Bumilleria sp. SAG 2160_1.77
ASV_176_EF431849.1_Bumilleriopsis sp. SAG 33.93_1.73
ASV_89_EF431849.1_Bumilleriopsis sp. SAG 33.93_1.68
ASV_73_EF431849.1_Bumilleriopsis sp. SAG 33.93_1.81
ASV_122_EF455960.1_Bumilleriopsis sp. SAG 34.93_1.81
ASV_131_EF455960.1_Bumilleriopsis sp. SAG 34.93_1.83
ASV_153_EF455960.1_Bumilleriopsis sp. SAG 34.93_1.71
ASV_72_EF455960.1_Bumilleriopsis sp. SAG 34.93_1.85
ASV_81_MK909757.1_Chlorellidium tetrabotrys_1.31
ASV_68_MK909757.1_Chlorellidium tetrabotrys_1.85
ASV_124_MK792452.1_Heterococcus leptosiroides_1.66
ASV_144_MK792452.1_Heterococcus leptosiroides_1.68
ASV_123_MK792452.1_Heterococcus leptosiroides_1.58
ASV_115_JX681222.1_Heterococcus mainxii_1.78
ASV_112_JN573801.1_Heterococcus sp._1.18
ASV_132_JN573801.1_Heterococcus sp._1.47
ASV_106_JN573801.1_Heterococcus sp._1.5
ASV_82_JN573801.1_Heterococcus sp._1.38
ASV_107_KM052781.1_Heterococcus sp. LCR-Ant10_2_1.41
ASV_121_KM052781.1_Heterococcus sp. LCR-Ant10_2_1.73
ASV_74_KM052781.1_Heterococcus sp. LCR-Ant10_2_1.68
ASV_69_MK804169.1_Heterothrix mucicola_1.85
ASV_142_AF064744.1_Mischococcus sphaerocephalus_1.46
ASV_166_MK909753.1_Pleurochloris meiringensis_1.78
ASV_4_AY682432.1_Tribonema regulare_1.73
ASV_86_KF598748.1_Vaucheria aversa_1.26
ASV_80_AF476945.1_Vaucheria frigida_1.85
ASV_9_AB266740.1_Vaucheria frigida_1.7
ASV_3_AB266740.1_Vaucheria frigida_1.73
ASV_1_AB266740.1_Vaucheria frigida_1.7
ASV_11_AF476954.1_Vaucheria hamata_1.85
ASV_110_AF476955.1_Vaucheria prona_1.85
ASV_213_AJ874702.1_Vaucheria terrestris_1.68
ASV_125_AJ874702.1_Vaucheria terrestris_1.85
ASV_16_AJ874702.1_Vaucheria terrestris_1.73
ASV_62_MK792448.1_Xanthonema bristolianum_1.85
ASV_15_MK792449.1_Xanthonema exile_1.85
ASV_133_AY682398.1_Xanthonema hormidioides_1.85
ASV_101_EF455974.1_Xanthonema montanum_1.8
ASV_49_EF455974.1_Xanthonema montanum_1.8
ASV_70_EF426796.1_Xanthonema sp. SAG 2179_1.83
ASV_60_EF426796.1_Xanthonema sp. SAG 2179_1.83
ASV_17_EF426796.1_Xanthonema sp. SAG 2179_1.83
ASV_7_EF426796.1_Xanthonema sp. SAG 2179_1.85
ASV_41_EF426794.1_Xanthonema sp. SAG 2192_1.63
ASV_31_EF426794.1_Xanthonema sp. SAG 2192_1.65

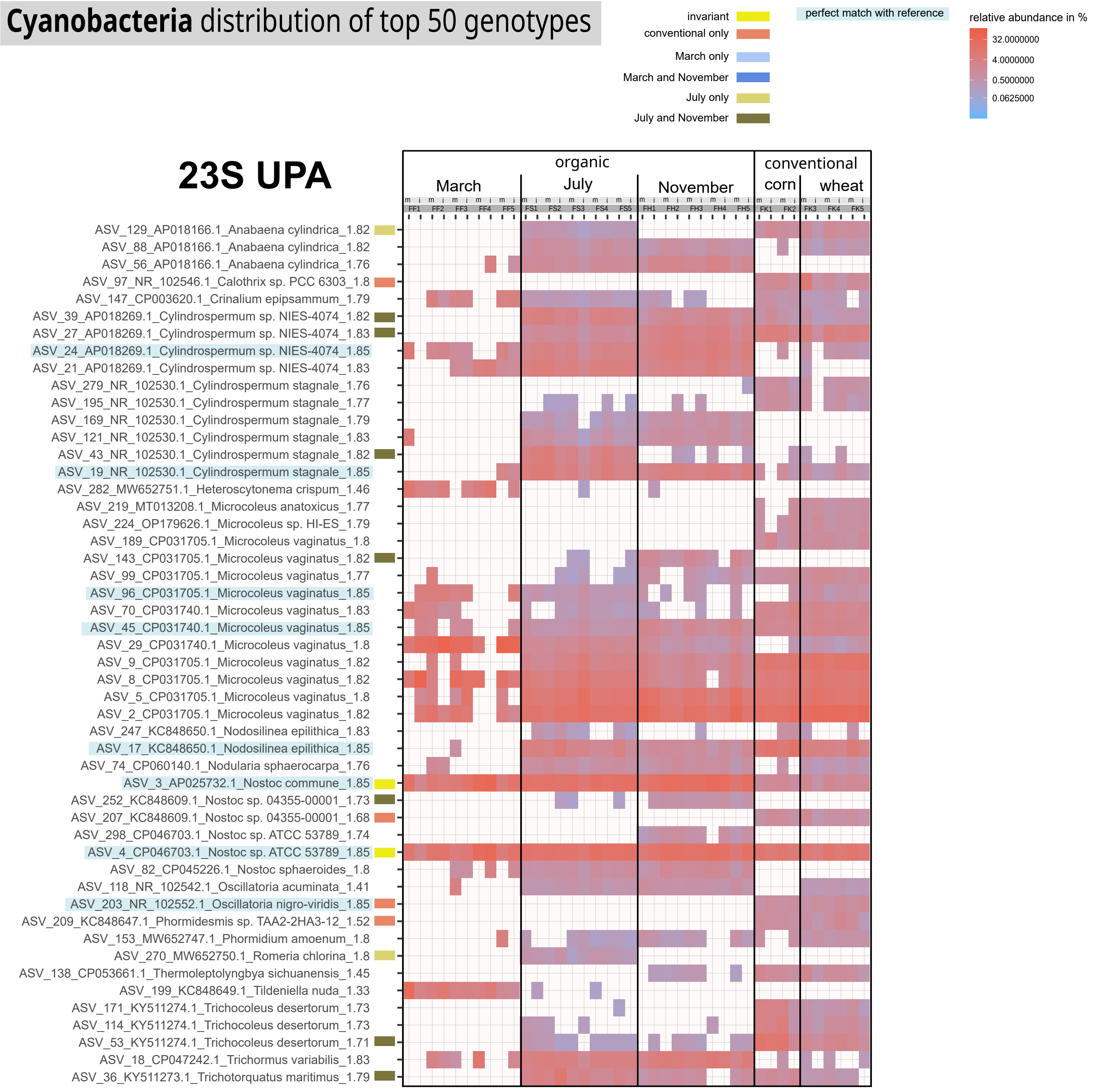
Cyanobacteria distribution of top 50 genotypes
invariant
conventional only
March only
March and November
July only
July and November
perfect match with reference
relative abundance in %
32.0000000
4.0000000
0.5000000
0.0625000
23S UPA
organic
conventional
March
July
November
corn
wheat
FF1 FF2 FF3 FF4 FF5 FS1 FS2 FS3 FS4 FS5 FH1 FH2 FH3 FH4 FH5 FK1 FK2 FK3 FK4 FK5
ASV_129_AP018166.1_Anabaena cylindrica_1.82
ASV_88_AP018166.1_Anabaena cylindrica_1.82
ASV_56_AP018166.1_Anabaena cylindrica_1.76
ASV_97_NR_102546.1_Calothrix sp. PCC 6303_1.8
ASV_147_CP003620.1_Crinalium epipsammum_1.79
ASV_39_AP018269.1_Cylindrospermum sp. NIES-4074_1.82
ASV_27_AP018269.1_Cylindrospermum sp. NIES-4074_1.83
ASV_24_AP018269.1_Cylindrospermum sp. NIES-4074_1.85
ASV_21_AP018269.1_Cylindrospermum sp. NIES-4074_1.83
ASV_279_NR_102530.1_Cylindrospermum stagnale_1.76
ASV_195_NR_102530.1_Cylindrospermum stagnale_1.77
ASV_169_NR_102530.1_Cylindrospermum stagnale_1.79
ASV_121_NR_102530.1_Cylindrospermum stagnale_1.83
ASV_43_NR_102530.1_Cylindrospermum stagnale_1.82
ASV_19_NR_102530.1_Cylindrospermum stagnale_1.85
ASV_282_MW652751.1_Heteroscytonema crispum_1.46
ASV_219_MT013208.1_Microcoleus anatoxicus_1.77
ASV_224_OP179626.1_Microcoleus sp. HI-ES_1.79
ASV_189_CP031705.1_Microcoleus vaginatus_1.8
ASV_143_CP031705.1_Microcoleus vaginatus_1.82
ASV_99_CP031705.1_Microcoleus vaginatus_1.77
ASV_96_CP031705.1_Microcoleus vaginatus_1.85
ASV_70_CP031740.1_Microcoleus vaginatus_1.83
ASV_45_CP031740.1_Microcoleus vaginatus_1.85
ASV_29_CP031740.1_Microcoleus vaginatus_1.8
ASV_9_CP031705.1_Microcoleus vaginatus_1.82
ASV_8_CP031705.1_Microcoleus vaginatus_1.82
ASV_5_CP031705.1_Microcoleus vaginatus_1.8
ASV_2_CP031705.1_Microcoleus vaginatus_1.82
ASV_247_KC848650.1_Nodosilinea epilithica_1.83
ASV_17_KC848650.1_Nodosilinea epilithica_1.85
ASV_74_CP060140.1_Nodularia sphaerocarpa_1.76
ASV_3_AP025732.1_Nostoc commune_1.85
ASV_252_KC848609.1_Nostoc sp. 04355-00001_1.73
ASV_207_KC848609.1_Nostoc sp. 04355-00001_1.68
ASV_298_CP046703.1_Nostoc sp. ATCC 53789_1.74
ASV_4_CP046703.1_Nostoc sp. ATCC 53789_1.85
ASV_82_CP045226.1_Nostoc sphaeroides_1.8
ASV_118_NR_102542.1_Oscillatoria acuminata_1.41
ASV_203_NR_102552.1_Oscillatoria nigro-viridis_1.85
ASV_209_KC848647.1_Phormidesmis sp. TAA2-2HA3-12_1.52
ASV_153_MW652747.1_Phormidium amoenum_1.8
ASV_270_MW652750.1_Romeria chlorina_1.8
ASV_138_CP053661.1_Thermoleptolyngbya sichuanensis_1.45
ASV_199_KC848649.1_Tildeniella nuda_1.33
ASV_171_KY511274.1_Trichocoleus desertorum_1.73
ASV_114_KY511274.1_Trichocoleus desertorum_1.73
ASV_53_KY511274.1_Trichocoleus desertorum_1.71
ASV_18_CP047242.1_Trichormus variabilis_1.83
ASV_36_KY511273.1_Trichotorquatus maritimus_1.79

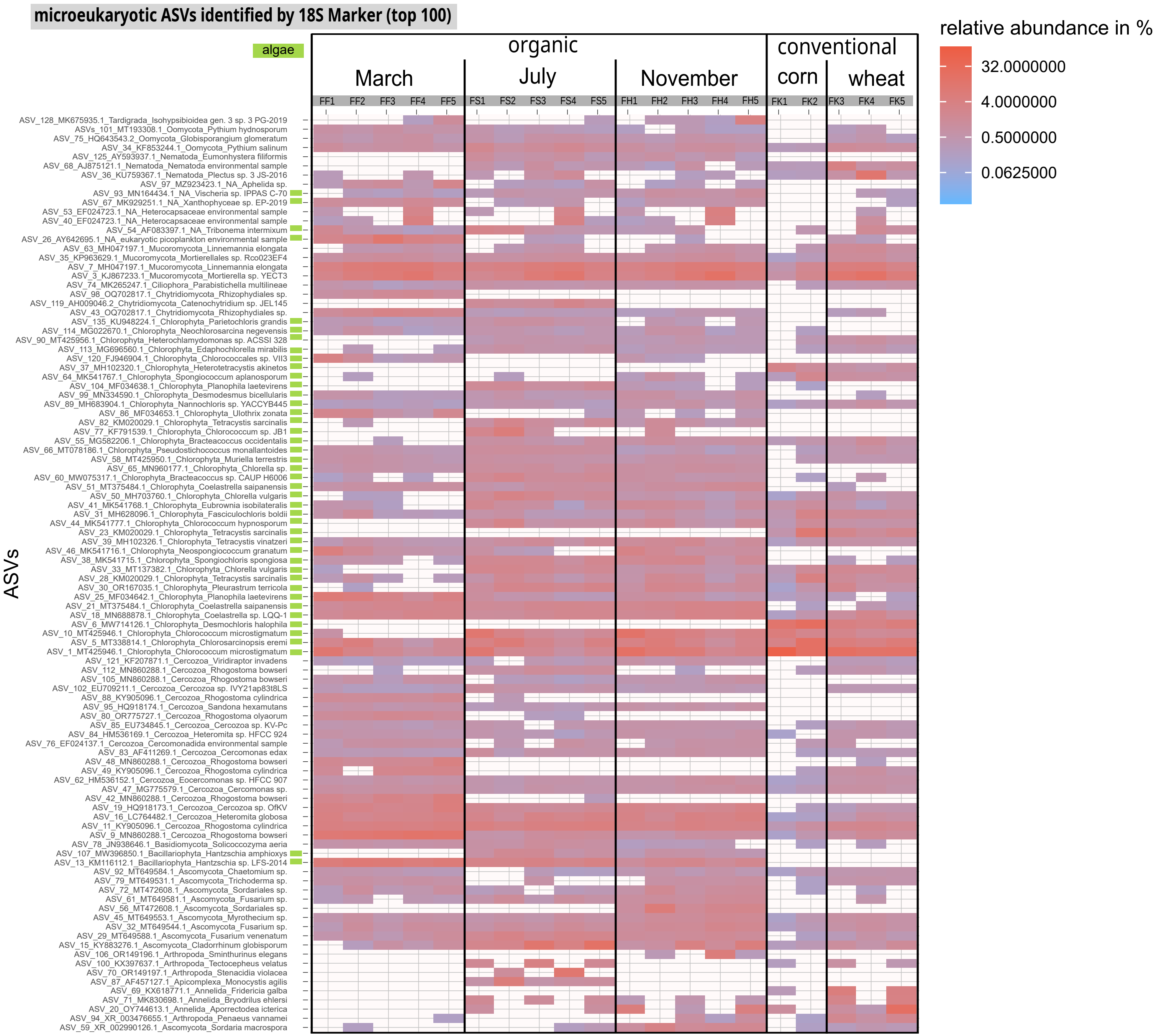
microeukaryotic ASVs identified by 18S Marker (top 100)
algae
organic
conventional
March
July
November
corn
wheat
FF1
FF2
FF3
FF4
FF5
FS1
FS2
FS3
FS4
FS5
FH1
FH2
FH3
FH4
FH5
FK1
FK2
FK3
FK4
FK5
relative abundance in %
32.0000000
4.0000000
0.5000000
0.0625000
ASVs
ASV_128_MK675935.1_Tardigrada_Isohypsibioidea gen. 3 sp. 3 PG-2019
ASVs_101_MT193308.1_Oomycota_Pythium hydnosporum
ASV_75_HQ643543.2_Oomycota_Globisporangium glomeratum
ASV_34_KF853244.1_Oomycota_Pythium salinum
ASV_125_AY593937.1_Nematoda_Eumonhystera filiformis
ASV_68_AJ875121.1_Nematoda_Nematoda environmental sample
ASV_36_KU759367.1_Nematoda_Plectus sp. 3 JS-2016
ASV_97_MZ923423.1_NA_Aphelida sp.
ASV_93_MN164434.1_NA_Vischeria sp. IPPAS C-70
ASV_67_MK929251.1_NA_Xanthophyceae sp. EP-2019
ASV_53_EF024723.1_NA_Heterocapsaceae environmental sample
ASV_40_EF024723.1_NA_Heterocapsaceae environmental sample
ASV_54_AF083397.1_NA_Tribonema intermixum
ASV_26_AY642695.1_NA_eukaryotic picoplankton environmental sample
ASV_63_MH047197.1_Mucoromycota_Linnemannia elongata
ASV_35_KP963629.1_Mucoromycota_Mortierellales sp. Rco023EF4
ASV_7_MH047197.1_Mucoromycota_Linnemannia elongata
ASV_3_KJ867233.1_Mucoromycota_Mortierella sp. YECT3
ASV_74_MK265247.1_Ciliophora_Parabistichella multilineae
ASV_98_OQ702817.1_Chytridiomycota_Rhizophydiales sp.
ASV_119_AH009046.2_Chytridiomycota_Catenochytridium sp. JEL145
ASV_43_OQ702817.1_Chytridiomycota_Rhizophydiales sp.
ASV_135_KU948224.1_Chlorophyta_Parietochloris grandis
ASV_114_MG022670.1_Chlorophyta_Neochlorosarcina negevensis
ASV_90_MT425956.1_Chlorophyta_Heterochlamydomonas sp. ACSSI 328
ASV_113_MG696560.1_Chlorophyta_Edaphochlorella mirabilis
ASV_120_FJ946904.1_Chlorophyta_Chlorococcales sp. VII3
ASV_37_MH102320.1_Chlorophyta_Heterotetracystis akinetos
ASV_64_MK541767.1_Chlorophyta_Spongiococcum aplanosporum
ASV_104_MF034638.1_Chlorophyta_Planophila laetevirens
ASV_99_MN334590.1_Chlorophyta_Desmodesmus bicellularis
ASV_89_MH683904.1_Chlorophyta_Nannochloris sp. YACCYB445
ASV_86_MF034653.1_Chlorophyta_Ulothrix zonata
ASV_82_KM020029.1_Chlorophyta_Tetracystis sarcinalis
ASV_77_KF791539.1_Chlorophyta_Chlorococcum sp. JB1
ASV_55_MG582206.1_Chlorophyta_Bracteacoccus occidentalis
ASV_66_MT078186.1_Chlorophyta_Pseudostichococcus monallantoides
ASV_58_MT425950.1_Chlorophyta_Muriella terrestris
ASV_65_MN960177.1_Chlorophyta_Chlorella sp.
ASV_60_MW075317.1_Chlorophyta_Bracteacoccus sp. CAUP H6006
ASV_51_MT375484.1_Chlorophyta_Coelastrella saipanensis
ASV_50_MH703760.1_Chlorophyta_Chlorella vulgaris
ASV_41_MK541768.1_Chlorophyta_Eubrownia isobilateralis
ASV_31_MH628096.1_Chlorophyta_Fasciculochloris boldii
ASV_44_MK541777.1_Chlorophyta_Chlorococcum hypnosporum
ASV_23_KM020029.1_Chlorophyta_Tetracystis sarcinalis
ASV_39_MH102326.1_Chlorophyta_Tetracystis vinatzeri
ASV_46_MK541716.1_Chlorophyta_Neospongiococcum granatum
ASV_38_MK541715.1_Chlorophyta_Spongiochloris spongiosa
ASV_33_MT137382.1_Chlorophyta_Chlorella vulgaris
ASV_28_KM020029.1_Chlorophyta_Tetracystis sarcinalis
ASV_30_OR167035.1_Chlorophyta_Pleurastrum terricola
ASV_25_MF034642.1_Chlorophyta_Planophila laetevirens
ASV_21_MT375484.1_Chlorophyta_Coelastrella saipanensis
ASV_18_MN688878.1_Chlorophyta_Coelastrella sp. LQQ-1
ASV_6_MW714126.1_Chlorophyta_Desmochloris halophila
ASV_10_MT425946.1_Chlorophyta_Chlorococcum microstigmatum
ASV_5_MT338814.1_Chlorophyta_Chlorosarcinopsis eremi
ASV_1_MT425946.1_Chlorophyta_Chlorococcum microstigmatum
ASV_121_KF207871.1_Cercozoa_Viridiraptor invadens
ASV_112_MN860288.1_Cercozoa_Rhogostoma bowseri
ASV_105_MN860288.1_Cercozoa_Rhogostoma bowseri
ASV_102_EU709211.1_Cercozoa_Cercozoa sp. IVY21ap83t8LS
ASV_88_KY905096.1_Cercozoa_Rhogostoma cylindrica
ASV_95_HQ918174.1_Cercozoa_Sandona hexamutans
ASV_80_OR775727.1_Cercozoa_Rhogostoma olyaorum
ASV_85_EU734845.1_Cercozoa_Cercozoa sp. KV-Pc
ASV_84_HM536169.1_Cercozoa_Heteromita sp. HFCC 924
ASV_76_EF024137.1_Cercozoa_Cercomonadida environmental sample
ASV_83_AF411269.1_Cercozoa_Cercomonas edax
ASV_48_MN860288.1_Cercozoa_Rhogostoma bowseri
ASV_49_KY905096.1_Cercozoa_Rhogostoma cylindrica
ASV_62_HM536152.1_Cercozoa_Eocercomonas sp. HFCC 907
ASV_47_MG775579.1_Cercozoa_Cercomonas sp.
ASV_42_MN860288.1_Cercozoa_Rhogostoma bowseri
ASV_19_HQ918173.1_Cercozoa_Cercozoa sp. OfKV
ASV_16_LC764482.1_Cercozoa_Heteromita globosa
ASV_11_KY905096.1_Cercozoa_Rhogostoma cylindrica
ASV_9_MN860288.1_Cercozoa_Rhogostoma bowseri
ASV_78_JN938646.1_Basidiomycota_Solicoccozyma aeria
ASV_107_MW396850.1_Bacillariophyta_Hantzschia amphioxys
ASV_13_KM116112.1_Bacillariophyta_Hantzschia sp. LFS-2014
ASV_92_MT649584.1_Ascomycota_Chaetomium sp.
ASV_79_MT649531.1_Ascomycota_Trichoderma sp.
ASV_72_MT472608.1_Ascomycota_Sordariales sp.
ASV_61_MT649581.1_Ascomycota_Fusarium sp.
ASV_56_MT472608.1_Ascomycota_Sordariales sp.
ASV_45_MT649553.1_Ascomycota_Myrothecium sp.
ASV_32_MT649544.1_Ascomycota_Fusarium sp.
ASV_29_MT649588.1_Ascomycota_Fusarium venenatum
ASV_15_KY883276.1_Ascomycota_Cladorrhinum globisporum
ASV_106_OR149196.1_Arthropoda_Sminthurinus elegans
ASV_100_KX397637.1_Arthropoda_Tectocepheus velatus
ASV_70_OR149197.1_Arthropoda_Stenacidia violacea
ASV_87_AF457127.1_Apicomplexa_Monocystis agilis
ASV_69_KX618771.1_Annelida_Fridericia galba
ASV_71_MK830698.1_Annelida_Bryodrilus ehlersi
ASV_20_OY744613.1_Annelida_Aporrectodea icterica
ASV_94_XR_003476655.1_Arthropoda_Penaeus vannamei
ASV_59_XR_002990126.1_Ascomycota_Sordaria macrospora
